# Targeted and non-targeted analyses of per-and polyfluoroalkyl substances in newborn dried blood spots and risk of childhood acute lymphoblastic leukemia

**DOI:** 10.1038/s41370-026-00891-6

**Published:** 2026-04-14

**Authors:** Veronica M. Vieira, Sheng Liu, Libby M. Morimoto, Jeremy Koelmel, Natalie R. Binczewski, Joseph L. Wiemels, Xiaomei Ma, Krystal J. Godri Pollitt, Catherine Metayer

**Affiliations:** 1 Department of Environmental and Occupational Health, Joe C. Wen School of Population & Public Health, University of California, Irvine, CA USA; 2 Department of Environmental Health Sciences, Yale University, New Haven, CT USA; 3 Division of Epidemiology, School of Public Health, University of California, Berkeley, CA USA; 4 Center for Genetic Epidemiology, Department of Population and Public Health Sciences, University of Southern California Keck School of Medicine, Los Angeles, CA USA; 5 Department of Chronic Disease Epidemiology, Yale School of Public Health, New Haven, CT USA

**Keywords:** PFAS, Childhood leukemia, Epidemiology, Dried blood spots, Fluoromatch

## Abstract

**Background:**

Per- and polyfluoroalkyl substances (PFAS) have carcinogenic potential but are understudied in relation to childhood cancers.

**Objective:**

We examined associations between targeted and non-targeted PFAS measured in newborn dried blood spots (DBS) and the risk of childhood acute lymphoblastic leukemia (ALL) in Los Angeles County, California, accounting for maternal and child characteristics.

**Methods:**

ALL cases (*n* = 125) diagnosed before age 18 years during 2000–2015 and controls (*n* = 219) were selected from a registry-based study using stratified sampling based on birth year and birth address within a PFAS-contaminated water district according to the USEPA Third Unregulated Contaminant Monitoring Rule. We calculated design-based odds ratios (OR) and 95% confidence intervals (CI) for the effect of PFAS exposures, independently and adjusting for other PFAS. We also conducted non-linear and stratified analyses.

**Results:**

Of the 17 PFAS quantified using targeted analysis, perfluorooctanoic acid (PFOA) and perfluorooctanesulfonic acid (PFOS) had the highest mean concentrations in DBS, with 4690 and 10,307 pg/g dried blood among cases compared to 4245 and 8142 pg/g dried blood among controls, respectively. The highest risks were observed for the 4th exposure quartile compared with the 1st quartile (PFOA OR = 1.56, CI: 0.42, 5.73; PFOS OR = 1.64, CI: 0.44, 6.14). In non-linear statistical analyses of joint PFOA and PFOS exposures adjusted for other detected PFAS, we also found that ALL risk increased with increasing levels of log2-PFOA and log2-PFOS. Non-targeted analysis identified 26 additional PFAS, for which elevated risk of childhood ALL was associated with a doubling of C_4_HF_7_O_3_ exposure (OR = 5.04, CI: 1.08, 23.63) and the highest quartile of C_10_HF_19_O_5_ exposure (OR = 5.20, CI: 1.15, 23.56). Associations were generally stronger among non-Hispanic participants compared to Hispanic participants, but these analyses were limited by small sample sizes and should be considered exploratory.

**Significance:**

There was some suggestion that high PFOA and PFOS exposures measured at birth, as well as certain PFAS detected by non-targeted approaches, were related to childhood ALL risk.

**Impact statement:**

This study of childhood ALL in Los Angeles County, California found increased risk among children with the highest PFOA and PFOS levels measured in neonatal dried blood spots. These risks were stronger among non-Hispanic children, and the greatest risk was observed for joint PFOA and PFOS exposure adjusting for other detected PFAS and maternal/child characteristics. We also observed possible associations with PFAS discovered by non-targeted analysis. Our results highlight the utility of dried blood spots as a matrix for assessing early life exposures as well as the value of hybrid targeted and non-targeted approaches to measure PFAS in health studies.

## Introduction

Per- and polyfluoroalkyl substances (PFAS) are a diverse class of fluorinated chemicals with thousands of chemical structures that have been widely used since the 1950s because of their resistance to heat and unique surfactant properties [1–3]. They are incorporated into products such as personal care products, non-stick cookware, water-resistant apparel, disposable food packaging, stain-resistant treatments for carpet and furniture, and aqueous film forming firefighting foam (AFFF) [4–8]. PFAS, particularly long chained compounds, are persistent in the environment and bioaccumulate [9, 10]. While several longer chained PFAS compounds—e.g., perfluorooctanoic acid (PFOA) and perfluorooctanesulfonic acid (PFOS)—were phased out in the United States (U.S.) during 2000–2015, some are still produced internationally and may be present in imported products [4, 5]. These longer chained PFAS compounds are being replaced by new shorter chained compounds. Although plasma concentrations of PFAS are declining in the U.S. because of the phase-out and the shorter half-lives of newer compounds, PFAS have been detected in the blood of 98% of the U.S. population, with legacy PFAS still detectable in blood from young adults born after the phase-out [4, 11].

There are numerous subclasses of PFAS, including perfluoroalkyl carboxylic acids (PFCAs), perfluoroalkyl sulfonic acids (PFSAs), fluorotelomer alcohols (FTOHs), and polyfluoroalkyl phosphoric acid esters (PAPs). Perfluoroalkyl acids (PFAAs), which include the PFCAs and PFSAs, are the most persistent PFAS compounds. Some PFAAs, particularly PFOS and PFOA, have long half-lives in humans, and exposure to these compounds has been associated with adverse health impacts [4]. Other PFAS, such as the FTOHs and PAPs, are less persistent due to their physicochemical properties; however, these compounds can degrade into the more persistent PFAAs and often are referred to as PFAA precursors [8]. Other precursors include perfluorooctyl sulfonamides (FOSAs) and sulfonamidoethanols (FOSEs). Given the ubiquitous presence of PFAS, it is important to understand their health effects, especially among vulnerable populations like children.

Many PFAS are highly soluble in water, and PFAS exposure from drinking water is a serious concern. High levels of PFAS are still detected in drinking water throughout the US, including California, as documented by water test results (2013–2015) from EPA’s Third Unregulated Contaminant Monitoring Rule, UCMR3 [12]. Consumption of drinking water contaminated with PFAS, even at relatively low concentrations, can lead to elevated exposure [4, 13]. Dietary exposure to PFAS, via contaminated food and food packaging, and exposure from household dust have also been documented [4, 14, 15].

The incidence of several types of childhood cancers (i.e., leukemia, and cancers of the brain, testis, and thyroid) has increased in the last decades in the U.S. and other industrialized countries, suggestive of a role of environmental factors [16–19]. Studies of childhood leukemia have consistently reported associations with pre- and postnatal exposure to pesticides, paints, solvents, and air pollution [20], any of which are classified as possible, probable, or definite human carcinogens. Recently, our group reported increased risks of childhood acute lymphoblastic leukemia (ALL) with high levels of PFAS measured in home dust [21].

The objective of this study was to evaluate possible associations between PFAS, assessed using a combination of targeted and non-targeted approaches in newborn dried blood spots (DBS), and the risk of childhood ALL by leveraging a large California statewide study of childhood cancers. Los Angeles (LA) County was chosen as the study area because of its large and diverse population and because it contained the greatest number of California public water districts with PFAS detections in UCMR3 (*n* = 10). Using a survey design to maximize exposure sampling and novel PFAS analytic methods, we can contribute important new insight towards the understanding of childhood health effects of a wider range of PFAS chemicals.

## Methods

### Study population

Cases (*n* = 125) of childhood ALL and cancer-free controls (*n* = 219) born in LA County California, during 2000–2015 were identified from the California Linkage Study of Early-onset Cancers (CALSEC). Details about the design of CALSEC have been published previously [22–24]. Of note, CALSEC contains information on the availability of newborn DBS collected approximately within 36 h after birth and archived by the California Biobank Program. In addition, maternal residential addresses at the time of birth were geocoded and linked to UCMR3 public water districts [25]. Participants with available DBS were selected using a stratified sampling approach based on case status, birth year, and whether their birth address was within a public water district with detected PFOA or PFOS in UCMR3. We oversampled children born in a contaminated water district, selecting all available cases and stratifying control sampling so that a similar number of controls were selected for each birth year and water district strata. Children born outside of contaminated water districts were sampled so that similar numbers of cases and controls across the temporal range of birth years and spatial extent of LA County were selected. Sampling weights were calculated for each participant and accounted for in the statistical analysis. Cases were diagnosed with ALL at the age of 0–14 years and were identified using the International Classification of Childhood Cancer (ICCC) site group 1 A codes. The study protocol was approved by the institutional review boards at the Universities of California in Irvine and Berkeley, and the California Department of Public Health.

### PFAS measurements

#### Extraction

DBS cards for study participants were shipped by the California Biobank Program to the Pollitt Lab at Yale University for PFAS analysis. A quarter of each DBS sample (diameter 13 mm) was cut out and placed in a 5 mL polypropylene Eppendorf tube. A paired card blank with similar area was also cut out from the same DBS card and placed in another tube to be analyzed alongside the DBS sample. All DBS and card blank samples were weighed by an analytical balance (Mettler-Toledo XS64), and their areas were measured by ImageJ. Before extraction, methanolic PFAS internal standards (10 pg/µL) were spiked on each DBS and card blank for the accurate quantification of PFAS. One extraction blank sample with no DBS or card blank in the Eppendorf tube was introduced for every extraction batch to monitor background PFAS contamination that might be introduced during extraction. One extraction spike sample was also included, where methanolic PFAS native standards (10 pg/µL) were used in the place of the DBS or card blank sample to ensure desired extraction efficiency was achieved.

All samples were treated with methanol (1 mL) and aggressively shaken with three stainless steel beads in a tissue homogenizer for 3 min at 1600 rpm to break down the DBS card. Samples were then sonicated for 10 min and centrifuged at 3660 rpm for 15 min. The supernatant (500 µL) from each sample was collected and transferred to a new 2 mL Eppendorf tube. Additional methanol (500 µL) was then added to samples and the extraction-sonication-centrifugation cycle was repeated twice. Supernatant from three cycles were combined (1.5 mL total) and filtered by passing through an Agilent Bond Elut C18 SPE cartridge and methanol in the resulting solution was evaporated under a nitrogen blow until complete dryness. Dried samples were then reconstituted with a methanol/water mixture (50:50, v/v, 100 µL).

To remove any insoluble components, all reconstituted samples were subject to an additional round of sonication for 10 min and were centrifuged at 15,000 rpm for 15 min. The supernatant (90 µL) was collected from each sample and transferred to a polypropylene LC autosampler vial and stored at −20 °C until analysis. Laboratory staff who processed and analyzed all samples were blinded to case–control status or any other characteristics of the subjects.

### Targeted and non-targeted PFAS analysis

Extracts were analyzed on an Agilent 1290 Infinity liquid chromatograph coupled to an Agilent 6546 quadrupole-time-of-flight mass spectrometer (LC-QTOF-MS) in negative electrospray ionization mode. All samples (10 µL) were injected onto the LC with an InfinityLab Poroshell 120 EC C18 analytical column (2.1 mm × 100 mm × 2.7 µm; Agilent) preceded by a SecurityGuard C18 Guard Cartridge (4 mm × 2 mm I.D.; Phenomenex) and two Zorbax DIOL guard columns (4.6 mm × 12.5 mm × 6 µm; Agilent). Data-dependent acquisition mode was used with iterative exclusion over mass-to-charge (m/z) ratios between 118 and 1500 Da with fragmentation spectra collected at a fixed collision energy at 40 eV from 50 to 1500 Da. Dynamic exclusion was enabled so that a feature would be excluded after one spectrum collected and released after 0.5 min. The aqueous mobile phase (A) was 2.5 mM ammonium acetate dissolved in LC-MS grade water, and the organic mobile phase (B) was LC-MS grade methanol. LC binary pump flow rate was set at 0.4 mL/min, and the starting mobile phase composition was 75% A and 25% B from 0 to 1 min. From 1 min to 3 min, the organic mobile phase B percentage was increased from 25% to 75%. From 3 min to 8 min, B percentage further increased from 75% to 100% and was held at 100% until 12 min, when the end of the run was reached. The samples were analyzed along with a six-point calibration curve with native PFAS concentrations at 0.01, 0.05, 0.1, 0.5, 1, and 2 pg/µL.

Targeted PFAS identification and quantification for the panel of 24 PFAS (Supplemental Table S1) was performed using Agilent MassHunter Quant (QTOF) software. Based on extremely high levels in the measurements, PFHpA might have been introduced into the samples during sample collection, transport, or storage before identification, so was excluded from statistical analyses. Software peak integrations were refined with manual inspection for every PFAS native and internal standards. Each PFAS in the targeted panel was paired with an internal standard of the same or similar structure with the closest retention time. Response ratios (area ratios between native and internal standards) were calculated and used for PFAS quantification.

A normalization workflow was adopted after raw measurements in pg/µL were obtained. Concentrations were normalized by subtracting theoretical PFAS levels in the paper card and by using grams of dried blood as the denominator in the reporting unit, calculated from the weight of DBS and the density of the card paper. A detailed description of the normalization workflow can be found in Lin et al. [26]. Final PFAS concentrations were reported in nanograms of PFAS in grams of dried blood. Limits of detection (LOD) for the targeted PFAS are presented in Supplemental Table S1.

Non-targeted approaches (NTA) were used to determine semi-quantitative exposure estimates for structures that were similar to internal standards by normalizing peak areas of those features against those of internal standards (1 ppb in final extract). Mass-labeled, non-H-substituted PFCAs were assigned to H-substituted PFCAs as corresponding internal standards depending on the carbon chain lengths; similarly, mass-labeled PFSAs were assigned to the two PFSA-ethers. Supplemental Table S2 includes information on the internal standard used for each of the NTA PFAS identified by this approach. Peak areas for NTA PFAS features, and their corresponding internal standards were integrated in the Agilent’s MassHunter Quantitative analysis software, and relative concentrations were calculated by the response ratio between the two. All PFAS concentrations were then normalized to nanograms of PFAS in grams of dried blood, using the same blank filtering and normalization workflow as described earlier for the targeted PFAS identifications, including unit conversion and background subtraction.

For identification of other non-targeted PFAS that were not similar to internal standards, Agilent’s Explorer software was used for peak picking. Centroid data files were extracted for molecular features using the parameters in Supplemental Table S3 and the features were retention-time aligned to correct for any retention time drifts across different analysis batches. Peak areas were integrated for all extracted features and normalized against the sum of responses of internal standards (M5Perfluoro-n-pentanoic acid, M5Perfluoro-n-hexanoic acid, M4Perfluoro-n-heptanoic acid, M8Perfluoro-n-octanoic acid, M9Perfluoro-n-nonanoic acid, M6Perfluoro-n-decanoic acid, MPerfluoro-n-dodecanoic acid, M8Perfluoro-octane sulfonate) from that sample. Features were kept only if their normalized peak areas at 95-percentile level in actual samples were higher than two times of combined response of the average in blanks plus three times standard deviation. Features were grouped in series using FluoroMatch Modular7 [27, 28] after blank filtering based on their m/z, so that PFAS-like molecular features in the same homologous series with similar mass defect values could be identified. Data visualization was performed on FluoroMatch Visualizer using PowerBI [28]. PFAS homologous series were confirmed by manual inspection on their retention time versus m/z trend; MS/MS fragments; extracted ion chromatogram in samples and blanks; and theoretical isotope distribution based on predicted formula. Confirmed PFAS features from non-targeted analysis were subject to an additional step of blank filtering, and only those with normalized peak areas in DBS samples five times higher than their paired blank in at least one sample were kept.

### Statistical methods

Analyses were conducted using the survey package (version 4.4-2) in R statistical software (version 4.5.0) to model data from a complex survey design, with inverse-probability weighting and design-based standard errors [20, 29, 30]. We calculated the descriptive statistics for birth characteristics and sociodemographic variables and reported *p*-values from a design-based analysis of variance (ANOVA). For PFAS exposures, mean and standard deviation (SD) were calculated for continuous variables, and frequency (n) and percentage (%) of the participants for categorical variables. The correlation of PFAS chemicals was assessed using Spearman’s correlation coefficients, separately for targeted and non-targeted PFAS.

PFAS concentrations detected in more than 50% of samples (PFOS and PFOA measured using targeted analysis, and C_4_HF_7_O_3_, C_4_HF_9_O_4_S, C_5_HF_9_O_3_, C_9_H_2_F_16_O_2_, C_14_H_2_F_26_O_2_, C_15_H_2_F_28_O_2_, C_13_H_2_F_24_O_2_, C_10_HF_19_O_5_, and C_16_H_8_Cl_2_F_6_N_2_O_2_S identified by NTA) were log2-transformed and included in models as both continuous variables and categorically in quartiles based on the distribution of the controls (Table 1). Four less-frequently detected targeted PFAS (PFHxS, FOSA, PFPeA, 6:2 FTS) were modeled as dichotomous variables ( ≥ LOD vs. <LOD). For PFOS and PFOA, values below the limit of quantification (LOQ) were randomly imputed to values between zero and the LOQ using the distribution of measured values for each PFAS [31]. Samples for which the concentrations in the card blanks were higher than in the DBS were considered contaminated and excluded from statistical analyses. For non-targeted PFAS without LOQ, samples with values below the limit of detection were not included in the statistical analyses.

**Table 1:** Summary of Imputed PFAS using Targeted and Non-Targeted Approaches Measured in Dried Blood Spots for Childhood Acute Lymphoblastic Leukemia (ALL) Cases and Controls in Los Angeles County, California (2000–2015).

Chemical	ALL Cases (*n* = 125)					Controls (*n* = 219)					*p*-value
	*n*	Mean (SD)	25th	50th	75th	*n*	Mean (SD)	25th	50th	75th	
Targeted Analyses											
PFOA	121	4689.6 (4280.2)	1644.0	3480.4	6578.8	214	4245.4 (4086.1)	1300.7	3151.3	5645.0	0.20
PFOS	124	10,307.3 (11901.8)	2472.1	6989.6	13,049.6	216	8142.2 (8015.5)	2058.1	5915.5	11786.9	0.09
PFPeA	119	840.6 (1088.2)	138.7	457.5	1112.2	204	891.5 (2006.5)	158.1	486.3	947.0	0.22
PFHxS	125	1169.1 (2083.2)	206.5	597.4	1237.7	219	1060.8 (1407.1)	277.0	666.8	1287.4	0.38
6:2 FTS	108	1314.8 (2362.7)	199.7	580.7	1643.0	193	1650.8 (3003.7)	199.7	580.7	1643.0	0.82
PFOSA	124	1685.7 (3397.2)	126.5	451.4	1577.3	218	1073.3 (1939.0)	103.4	388.6	992.4	0.15
Non-Targeted Analyses											
Semi-quantitative PFAS											
C_4_HF_7_O_3_	106	36,862.4 (51,863.9)	14,504.3	28,257.9	42,995.5	188	32,120.4 (25,788.2)	13,559.8	25,823.6	43,834.6	0.16
C_4_HF_9_O_4_S	107	152,072.6 (343,883.8)	53,436.6	92,918.3	168695.2	182	171,401.0 (187,725.3)	60,204.0	119,837.9	215,860.5	0.97
C_5_HF_9_O_3_	95	16,689.4 (21,432.5)	7433.8	12,646.1	18,579.4	173	15,470.3 (13,085.9)	6845.5	12,058.0	22,277.3	0.36
C_9_H_2_F_16_O_2_	101	32,474.6 (45,234.0)	14,748.9	21,662.3	33,352.4	164	29,225.9 (22,766.2)	14,742.4	23662.7	37,135.2	0.24
C_14_H_2_F_26_O_2_	82	49,743.3 (29,284.1)	31,104.2	40,332.6	64,011.0	125	43,113.4 (33,683.2)	23,780.0	32,951.8	46,174.8	0.19
C_15_H_2_F_28_O_2_	82	37,270.3 (19,956.0)	23,081.0	33,424.9	46,998.3	114	34,073.7 (22,515.8)	20,623.3	29,365.7	39,471.4	0.11
C_13_H_2_F_24_O_2_	66	54,528.7 (27886.7)	35,638.4	51,147.7	64,395.8	109	46,676.0 (28,519.3)	29,827.9	41,803.7	53,557.5	0.79
C_10_HF_19_O_5_	65	28,352.5 (65223.9)	3594.5	10,828.5	39,019.1	107	18,222.8 (31,494.7)	3738.6	9440.7	25,537.5	0.22
Other PFAS											
C_16_H_8_Cl_2_F_6_N_2_O_2_S	107	175,241.5 (157,747.4)	83,458.2	138,597.7	223,243.1	185	158,001.0 (128003.9)	60,541.1	119,313.6	22,1887.9	0.52

Targeted analyses and semi-quantitative non-targeted PFAS analyses measure pg chemical/ g dried blood. Non-targeted analyses of other PFAS measure normalized peak area of chemical. *P*-values are from design-based t-test.

Multivariable models included maternal age at birth ( < 25, ≥25 & < 35, ≥35 years) and insurance type used to pay for delivery of child (public, private, other). Associations between case/control status and additional covariates evaluated as possible confounders (birth year, age at DBS collection, sex assigned at birth, birthweight, gestational age, birth order, parity, delivery method, maternal history of miscarriage, child’s race and ethnicity, paternal age, parental education, mother’s country of birth, and median census tract house value) were not statistically significant in the survey design-based ANOVA (*p*-value > 0.05) and were not included in final models. Odds ratios (ORs) and 95% confidence intervals (CIs) were estimated using logistic regression with inverse probability weights and survey design-based standard errors to assess the associations between each PFAS and childhood ALL. We also fit a categorical model that mutually adjusted for all targeted PFAS; PFOA and PFOS levels were modeled in quartiles, and the detection of PFHxS, FOSA, PFPeA, and 6:2 FTS were modeled as dichotomous variables. For non-targeted PFAS, we fit a continuous model mutually adjusted for log2-transformed C_4_HF_7_O_3_, C_4_HF_9_O_4_S, C_9_H_2_F_16_O_2_, C_14_H_2_F_26_O_2_, C_15_H_2_F_28_O_2_, C_10_HF_19_O_5_, and C_16_H_8_Cl_2_F_6_N_2_O_2_S. C_5_HF_9_O_3_ and C_13_H_2_F_24_O_2_ were excluded due to collinearity with other PFAS based on Spearman’s correlation coefficients greater than 0.85 (Supplemental Fig. S1). All tests were two-sided with an alpha level of 0.05 for statistical significance.

To assess potential effect modification, we conducted exploratory analyses stratified by ethnicity (Hispanic versus non-Hispanic), age at diagnosis (≤2 years versus >2 years), sex assigned at birth (male versus female as indicated on the birth record), maternal birthplace (US-born versus foreign-born), and birth order (first born versus not first born) [32]. Our secondary analyses assessed non-linear associations between log2-transformed PFOS or PFOA and childhood ALL using natural spline models. Lastly, we fit a model with natural splines of both log2-transformed PFOS and PFOA, with and without adjustment for PFHxS, FOSA, PFPeA, and 6:2 FTS detection as dichotomous variables, to estimate the non-linear joint associations with PFOS and PFOA [21]. We did not fit a mixture model with non-targeted PFAS as the number of participants with detectable levels of all non-targeted PFAS was small (*n* = 96).

## Results

Summary statistics of imputed PFAS concentrations and the number of participants included in each analysis are reported in Table 1. Among the 23 PFAS quantified using targeted analysis, 17 were detected in DBS samples, but only 6 at >10% and 2 at >50% of the samples (*N* = 344, Supplemental Table S1). PFOS had the highest mean concentrations in DBS, with 10,307 pg/g dried blood among cases compared to 8142 pg/g dried blood among controls, followed by PFOA (4690 pg/g dried blood among cases compared to 4245 pg/g dried blood among controls). PFOS and PFOA were strongly correlated (*r* = 0.71). While average PFOS and PFOA concentrations differed for cases versus controls, the *p*-values did not reach statistical significance in crude comparisons via design-based t-tests (Table 1). There were 9 blood spots excluded from analyses for PFOA and 4 blood spots excluded for PFOS due to contamination of the blood spot cards.

The characteristics of cases and controls are presented in Table 2. Children with ALL were more likely to be male, to have older mothers, and to have private insurance for delivery compared to controls. All other birth and demographic characteristics were similar between cases and controls. Child’s year of birth and the median value of the home were strong predictors of PFOA and PFOS exposure (all *p* < 0.01), with children born in the early 2000s (versus more recent years) and with higher home values having higher levels (Supplemental Table S4). Children of parents who were older, with higher education, private insurance, and who were born in the U.S., also had higher PFOS levels. Non-Hispanic whites also had higher levels of PFOS and PFOA compared to other race/ethnicity groups, but differences were not statistically significant.

**Table 2:** Characteristics of Childhood Acute Lymphoblastic Leukemia (ALL) Cases and Controls in Los Angeles County, California (2000–2015).

	ALL Cases (*n* = 125)	Controls (*n* = 219)	P (χ^2^)^1^
Child’s birth year			0.99
2000–2004	50 (40.0%)	84 (38.4%)	
2005–2009	43 (34.4%)	72 (32.9%)	
2010–2015	32 (25.6%)	63 (28.7%)	
Child’s age at blood collection (hours)			0.21
<24	30 (24.0%)	61 (27.8%)	
≥24 & ≤48	73 (58.4%)	118 (53.9%)	
>48	22 (17.6%)	38 (17.4%)	
Missing	0 (0.0%)	2 (0.9%)	
Child’s sex			0.07
Male	67 (53.6%)	99 (45.2%)	
Female	58 (46.4%)	120 (54.8%)	
Child’s race/ethnicity			0.33
White	22 (17.6%)	44 (20.1%)	
Hispanic	92 (73.6%)	152 (69.4%)	
Other or unknown	11 (8.8%)	23 (10.5%)	
Birth order			0.62
First child	53 (42.4%)	88 (40.2%)	
Second child	41 (32.8%)	70 (32.0%)	
Third or more	31 (24.8%)	61 (27.9%)	
Total children ever born			0.54
One	53 (42.4%)	87 (39.7%)	
Two	40 (32.0%)	69 (31.5%)	
Three	18 (14.4%)	39 (17.8%)	
Four or more	14 (11.2%)	23 (10.5%)	
Missing	0 (0.0%)	1 (0.5%)	
Delivery method			0.83
Vaginal	87 (69.6%)	136 (62.1%)	
Cesarean	38 (30.4%)	83 (37.9%)	
History of miscarriage			0.61
No	106 (84.8%)	190 (86.7%)	
Yes	19 (15.2%)	28 (12.8%)	
Missing	0 (0.0%)	1 (0.5%)	
Maternal age (years)			0.04
<25	36 (28.8%)	72 (32.9%)	
≥25 & <35	65 (52.0%)	107 (48.9%)	
≥35	24 (19.2%)	40 (18.3%)	
Paternal age (years)			0.52
<25	18 (14.4%)	46 (21.0%)	
≥25 & <35	55 (44.0%)	93 (42.5%)	
≥35	52 (41.6%)	80 (36.5%)	
Maternal education			0.32
High school or less	62 (49.6%)	120 (54.8%)	
Some college or more	62 (49.6%)	94 (42.9%)	
Missing	1 (0.8%)	5 (2.3%)	
Paternal education			0.64
High school or less	58 (46.4%)	112 (51.1%)	
Some college or more	55 (44.0%)	83 (37.9%)	
Missing	12 (9.6%)	24 (11.0%)	
Insurance for delivery			0.03
Private	73 (58.4%)	105 (47.9%)	
Public	50 (40%)	102 (46.6%)	
Other	<5	12 (5.5%)	
Mother’s country of birth			0.72
Mexico	24 (19.2%)	45 (20.5%)	
US 50 states	74 (59.2%)	138 (63.0%)	
Other countries	23 (18.4%)	24 (11.0%)	
Unknown	4 (3.2%)	12 (5.5%)	
Median census tract house value ($1000)			0.68
≥10.0 & <142.8	26 (20.8%)	53 (24.2%)	
≥142.8 & <162.6	20 (16.0%)	53 (24.2%)	
≥162.6 & <197.7	40 (32.0%)	53 (24.2%)	
≥197.7 & ≤1000.0	38 (30.4%)	54 (24.7%)	
Missing	1 (0.8%)	6 (2.7%)	
Water district			0.85
Unexposed water district	73 (58.4%)	105 (47.9%)	
CA Water Service East Los Angeles	13 (10.4%)	14 (6.4%)	
City of Downey Water Department	7 (5.6%)	15 (6.8%)	
Golden State Water Co—Norwalk	7 (5.6%)	12 (5.5%)	
Montebello Land & Water Co	5 (4.0%)	11 (5.0%)	
Orchard Dale Water District	<5	6 (2.7%)	
Park Water Co—Bellflower/Norwalk	<5	11 (5.0%0	
City of Pico Rivera Water Department	<5	7 (3.2%)	
Pico Water District	<5	11 (5.0%)	
Santa Clarita Water Division	5 (4.0%)	15 (6.8%)	
Valencia Water Company	<5	12 (5.5%)	

*p*-values are from design-based ANOVA. Counts less than five for strata other than missing and unknown were suppressed.

Twenty-six PFAS were identified by NTA, including 13 H-substituted PFCAs, with chain lengths varying from 9 carbons to 22 carbons; and 5 H-substituted PFCAs with ether linkages and PFSAs with ether bonds, including shorter-chained PFAS alternatives such as perfluoro-3,6,9-trioxatridecanoic acid. These 18 PFAS identified by NTA had a similar structure to a commercially available mass-labeled internal standard, which enabled semi-quantified exposure estimates. For the other 8 PFAS that were identified, their exact structures could not be determined. One of those 8 was annotated as an aromatic PFAS based on fragmentation peaks and exact mass. It was not possible to annotate the other 7 novel PFAS, as their fragmentation pattern did not provide sufficient structural information, or because their precursor ions existed at low intensities on the mass spectrometer, and no MS/MS fragments were collected. A detailed table with identifications from the non-targeted analysis can be found in Supplemental Table S2.

Nine PFAS identified in the NTA were detected in >50% of the population. Average levels for PFAS identified in the NTA also differed for cases versus controls, but the *p*-values did not reach statistical significance (Table 1). C_16_H_8_Cl_2_F_6_N_2_O_2_S exposures were higher for children with earlier birth year and older parents (Supplemental Table S4). Levels of ether-linkage-containing PFCAs and PFSAs (C_4_HF_7_O_3_, C_4_HF_9_O_4_S, C_5_HF_9_O_3_, C_10_HF_19_O_5_) also tended to be higher for children of parents who were older, with higher education and private insurance, and notably, Asian children, and children of mothers born outside the US and Mexico (Supplemental Table S5). Conversely, longer chained H-substituted PFCAs (C_14_H_2_F_26_O_2_, C_15_H_2_F_28_O_2_, C_13_H_2_F_24_O) were higher among Hispanic children, children born in later years, born to younger parents, and children with US-born mothers (Supplemental Table S6). C_5_HF_9_O_3_ was strongly correlated with C_4_HF_7_O_3_ (*r* = 0.88) and C_4_HF_9_O_4_S (*r* = 0.80), and C_14_H_2_F_26_O_2_, C_15_H_2_F_28_O_2_ and C_13_H_2_F_24_O_2_ were strongly correlated with each other (Supplemental Table S6), particularly C_14_H_2_F_26_O_2_ and C_13_H_2_F_24_O_2_ (*r* = 0.88).

**Fig. 1:**
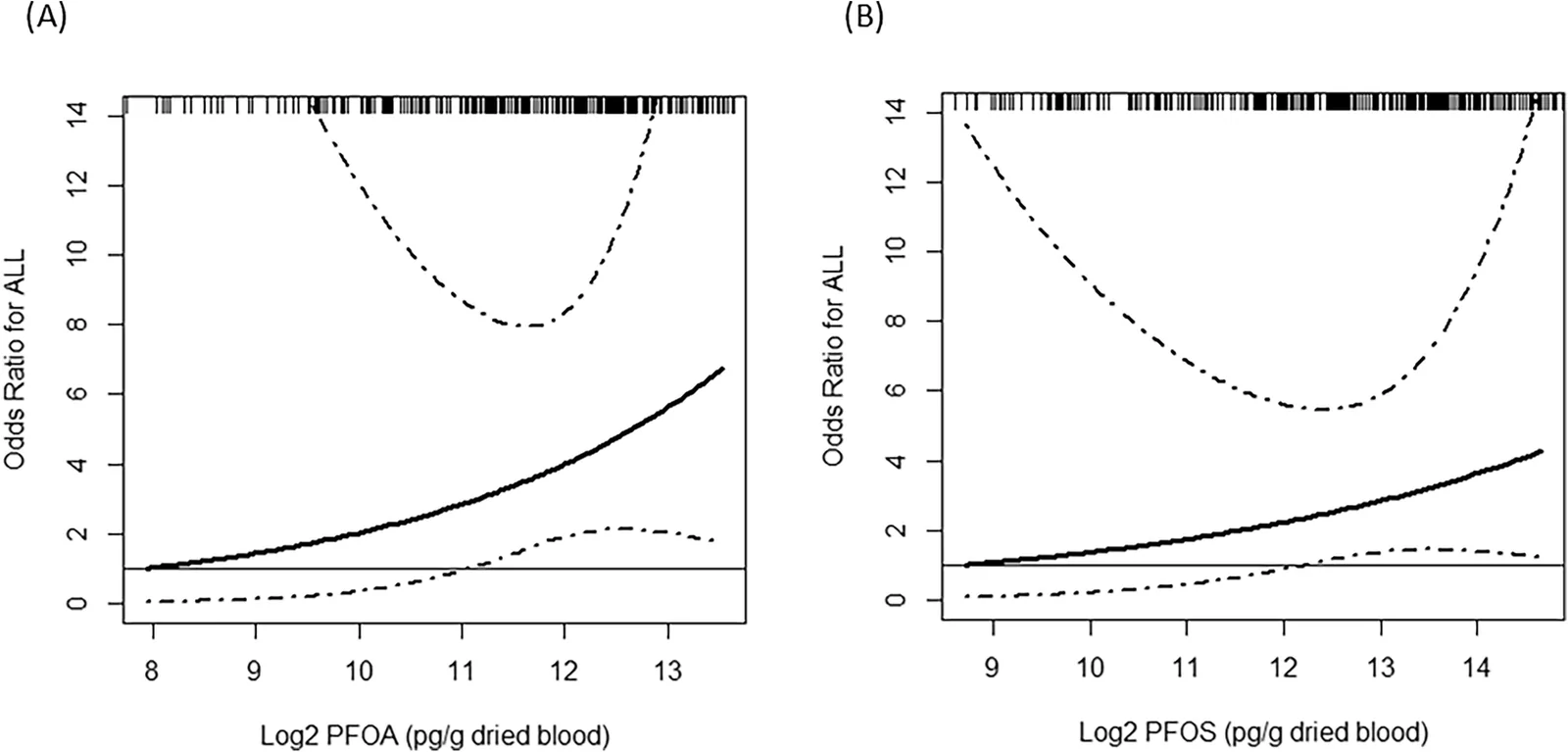
Odds Ratios (ORs, solid curve) and 95% Confidence Intervals (CI, dashed curve) for Associations between Targeted PFAS Measures in Dried Blood Spots and Childhood Acute Lymphoblastic Leukemia (ALL) in Los Angeles County, California (2000–2015). Separate models were fit with natural splines for (**A**) Log2 PFOA and (**B**) Log 2 PFOS. A rug of the measured data is shown at the top of the plots, and a line for OR = 1 is included at the bottom of the plots. The *p*-values for Log2 PFOA and Log2 PFOS based on permutation tests are 0.38 and 0.42, respectively.

In multivariable models for continuous linear PFAS measured by targeted analysis, increased PFOA and PFOS exposure resulted in increased risks of childhood ALL, albeit not statistically significant. The OR for a doubling of PFOA was 1.15 (95% CI: 0.84, 1.58) and the OR for a doubling of PFOS was 1.12 (95% CI: 0.85, 1.47) (Table 3). The categorical model for PFOS was suggestive of increasing effect across categories of increasing exposure, although the *p*-value for trend was not significant (*p* = 0.31). ORs for the 2nd, 3rd, and 4th quartiles compared with the 1st quartile were 0.65 (95% CI: 0.19, 2.23), 1.43 (95% CI: 0.42, 4.87), and 1.56 (95% CI: 0.42, 5.73), respectively. For PFOA, the highest risk was observed for the 4th quartile compared with the 1st quartile (OR = 1.64, 95% CI: 0.44, 6.14), followed by the 2nd and 3rd quartiles (OR = 1.10, 95% CI: 0.27, 4.42 and OR = 0.85, 95% CI: 0.23, 3.09, respectively). Results of the spline models for log2 PFOA (Fig. 1A, *p*-value = 0.38) and log2 PFOS (Fig. 1B, *p*-value = 0.42) were also not statistically significant and showed only slight deviations from a linear fit. Participants with detectable levels of PFPeA, PFHxS, and PFOSA had non-significant increased risk of childhood ALL compared to those who did not have detectable levels (OR = 1.75, 95% CI: 0.57, 5.40; OR = 1.99, 95% CI: 0.71, 5.62; and OR = 1.46, 95% CI: 0.54, 3.93, respectively). PFHxS detection resulted in a decrease in ALL risk (OR = 0.75, 95% CI: 0.24, 2.38).

**Table 3:** Odds Ratios (ORs) and 95% Confidence Intervals (CI) for Associations between Targeted PFAS Measures in Dried Blood Spots and Childhood Acute Lymphoblastic Leukemia (ALL) in Los Angeles County, California (2000–2015).

		Single PFAS Models			Mutually Adjusted PFAS Model	
Chemical (pg/g dried blood)	*n*	OR	95% CI	*p*-value	OR	95% CI
Continuous						
Log2 PFOA	335	1.15	0.84, 1.58	0.38		
Log2 PFOS	340	1.12	0.85, 1.47	0.41		
Categorical						
PFOA				0.56		
2.14–1365.89	79	---	---		---	---
1365.89–3136.46	82	1.10	0.27, 4.42		0.68	0.12, 3.71
3136.47–5645.01	84	0.85	0.23, 3.09		0.47	0.09, 2.48
5645.02–21569.07	90	1.64	0.44, 6.14		1.81	0.33, 9.88
PFOS				0.31		
9.13–2058.09	84	---	---		---	---
2058.10–5822.69	76	0.65	0.19, 2.23		1.07	0.24, 4.78
5822.70–11481.99	85	1.43	0.42, 4.87		2.54	0.38, 16.80
11482.00–43719.92	95	1.56	0.42, 5.73		1.63	0.17, 15.69
Binary (detect vs non-detect)						
PFPeA	323	1.75	0.57, 5.40	0.34	1.79	0.54, 5.93
PFHxS	344	1.99	0.71, 5.62	0.20	1.21	0.33, 4.43
6:2 FTS	301	0.75	0.24, 2.38	0.62	0.91	0.27, 3.06
PFOSA	342	1.46	0.54, 3.93	0.46	0.52	0.11, 2.54

Models were fit using a generalized linear model, with inverse-probability weighting and survey design-based standard errors. All models included maternal age at birth (<25, ≥25 & <35, ≥35 years) and insurance type used to pay for delivery of child (public, private, other). Quartiles are based on the distribution of controls. PFOA and PFOS were modeled categorically by quartiles and the detection of PFHxS, FOSA, PFPeA, and 6:2 FTS were modeled as dichotomous variables in the mutually adjusted models (*n* = 276, *p*-value = 0.69). *P*-values are from design-based ANOVA.

**Fig. 2:**
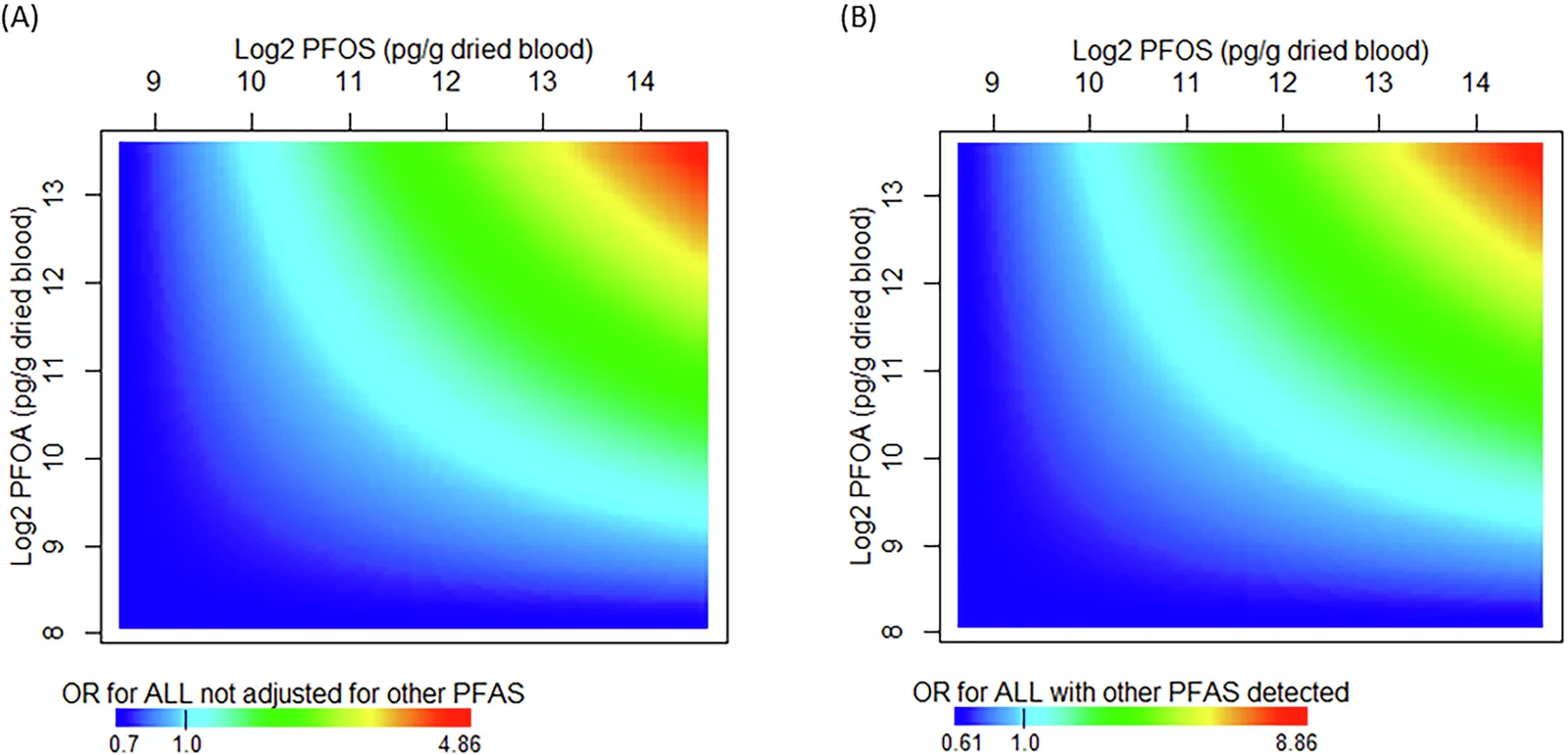
Odds Ratios (ORs) for Associations between Non-linear Joint Effects of Log2 PFOA and Log2 PFOS Measures in Dried Blood Spots and Childhood Acute Lymphoblastic Leukemia (ALL) in Los Angeles County, California (2000–2015). ALL ORs are predicted for children with varying levels of PFOA and PFOS exposure, **A** assuming the child’s mother is 25 to <35 years old with private health insurance and **B** further adjusted for detection of other targeted PFAS. The *p*-values for the joint effect of Log2 PFOA and Log2 PFOS based on permutation tests without and with adjustment for other targeted PFAS detections are 0.19 and 0.22, respectively.

Mutual adjustment for other targeted PFAS resulted in an increase in the effect estimates with categorical PFOS; ORs for the 2nd, 3rd, and 4th quartiles compared with the 1st quartile were 1.07 (95% CI: 0.24, 4.78), 2.54 (95% CI: 0.38, 16.80), and 1.63 (95% CI: 0.17, 15.69), respectively (Table 3). For PFOA, the risk observed for the 4th quartile compared with the 1st quartile was also higher compared to the single exposure model (OR = 1.81, 95% CI: 0.33, 9.88). The relationship with PFOSA detection flipped and was inversely related to ALL after mutual adjustment (OR = 0.52, 95% CI: 0.11, 2.54). In stratified analyses, effect estimates were consistently higher among non-Hispanic participants compared to Hispanic participants, with the exception of 6:2 FTS, but results were limited by smaller numbers (Supplemental Table S7). There were no other clear relationships between ALL and targeted PFAS stratified by diagnosis age, sex, and birth place strata (Supplemental Tables S8-S11). In non-linear statistical analyses of joint PFOA and PFOS exposures (Fig. 2), we observed the highest risk of ALL with the highest levels of log2-PFOA and log2-PFOS, especially in analyses adjusted for other PFAS detection (Fig. 2B).

For PFAS identified by NTA, increased risk of childhood ALL was observed per doubling of all PFAS except C_13_H_2_F_24_O_2_ (Table 4). Categorical analyses indicate a positive statistically significant trend with increasing quartiles of C_10_HF_19_O_5_ exposure compared to the 1st quartile (*p* = 0.01). C_4_HF_7_O_3_, C_9_H_2_F_16_O_2_, and C_14_H_2_F_26_O_2_ also showed non-significant increased ALL risk with increased exposure quartiles. In mutually adjusted models, C_4_HF_7_O_3_ was strongly associated with ALL (OR = 5.04, 95% CI: 1.08, 23.63). Risks associated with C_14_H_2_F_26_O_2_ and C_10_HF_19_O_5_ also remained elevated (Table 4). C_9_H_2_F_16_O_2_ was inversely related to ALL (OR = 0.33, 95% CI: 0.08, 1.39), but in the single non-targeted PFAS model, the association was flipped (OR = 1.26, 95% CI: 0.86, 1.86). The mutually-adjusted analysis was limited to only 86 participants with detectable measures of the seven PFAS (C_4_HF_7_O_3_, C_4_HF_9_O_4_S, C_9_H_2_F_16_O_2_, C_14_H_2_F_26_O_2_, C_15_H_2_F_28_O_2_, C_10_HF_19_O_5_, and C_16_H_8_Cl_2_F_6_N_2_O_2_S). Similar to the targeted PFAS analyses, effect estimates were usually higher among non-Hispanic participants compared to Hispanic, although again limited by smaller numbers (Supplemental Table S12). Statistically significantly increased ALL risk among non-Hispanic participants was observed for C_4_HF_7_0_3_, C_9_H_2_F_16_O_2_, and C_16_H_8_Cl_2_F_6_N_2_O_2_S. There were no obvious differences in ALL risks by other strata (Supplemental Tables S13–S16).

**Table 4:** Odds Ratios (ORs) and 95% Confidence Intervals (CI) for Associations between Non-Targeted PFAS Measures in Dried Blood Spots and Childhood Acute Lymphoblastic Leukemia (ALL) in Los Angeles County, California (2000–2015).

		Single PFAS Models			Mutually Adjusted PFAS Model	
Chemical (pg/g dried blood)	*n*	OR	95% CI	*p*-value	OR	95% CI
Continuous						
Log2 C_4_HF_7_O_3_	294	1.24	0.90, 1.71	0.32	5.04	1.08, 23.63
Log2 C_4_HF_9_O_4_S	289	1.06	0.84, 1.34	0.69	0.81	0.28, 2.36
Log2 C_5_HF_9_O_3_	268	1.27	0.91, 1.77	0.27		
Log2 C_9_H_2_F_16_O_2_	265	1.26	0.86, 1.86	0.38	0.33	0.08, 1.39
Log2 C_14_H_2_F_26_O_2_	207	1.60	0.98, 2.62	0.21	1.96	0.12, 33.30
Log2 C_15_H_2_F_28_O_2_	196	1.51	0.89, 2.56	0.26	1.19	0.02, 80.30
Log2 C_13_H_2_F_24_O_2_	175	0.99	0.51, 1.93	0.99		
Log2 C_10_HF_19_O_5_	172	1.23	0.96, 1.57	0.25	1.80	0.59, 5.53
Log2 C_16_H_8_Cl_2_F_6_N_2_O_2_S	292	1.17	0.82, 1.69	0.39	0.87	0.22, 3.43
Categorical						
C_4_HF_7_O_3_				0.87		
507.2–13,559.8	71	---	---			
13,559.8–25,823.6	70	1.23	0.30, 5.07			
25,823.7–43,834.6	80	1.56	0.35, 6.92			
43,834.7–495,276.0	73	1.79	0.38, 8.41			
C_4_HF_9_O_4_S				0.36		
2214.7–60,204.0	77	---	---			
60,204.1–119,837.9	76	1.62	0.36, 7.29			
119838.0–215,860.5	73	3.17	0.77, 13.10			
215,860.6–3,529,999.0	63	1.04	0.23, 4.75			
C_5_HF_9_O_3_				0.57		
160.9–6845.5	65	---	---			
6845.6–12,058.0	68	0.85	0.17, 4.19			
12,058.1–22,277.3	74	2.03	0.47, 8.67			
22,277.4–195,076.1	61	1.24	0.29, 5.39			
C_9_H_2_F_16_O_2_				0.64		
3391.8–14,742.4	66	---	---			
14,742.5–23,662.7	71	0.53	0.13, 2.23			
23,662.8–37,135.2	67	0.83	0.18, 3.87			
37,135.3–426,198.9	61	1.28	0.32, 5.05			
C_14_H_2_F_26_O_2_				0.18		
2603.3–23,780.0	42	---	---			
23,780.1 - 32,951.8	46	4.90	0.77, 31.19			
32,951.9 - 46,174.8	56	5.57	0.86, 36.07			
46,174.9–275,222.0	63	5.94	0.90, 39.35			
C_15_H_2_F_28_O_2_				0.56		
2201.0–20,623.3	45	---	---			
20,623.4–29,365.7	45	1.75	0.37, 8.32			
29,365.8–39,471.4	46	2.94	0.63, 13.81			
39,471.5–143,211.9	60	1.79	0.37, 8.72			
C_13_H_2_F_24_O_2_				0.89		
10,734.8–29,827.9	39	---	---			
29,828.0–41,803.7	41	2.05	0.31, 13.79			
41,803.8–53,557.5	36	1.11	0.17, 7.11			
53,557.6–21,2811.1	59	1.39	0.23, 8.26			
C_10_HF_19_O_5_				0.01		
486.6–3738.6	45	---	---			
3738.7–9440.7	36	0.20	0.02, 1.82			
9440.8–25,537.5	43	0.41	0.05, 3.55			
25,537.6–516,726.0	48	5.20	1.15, 23.56			
C_16_H_8_Cl_2_F_6_N_2_O_2_S				0.28		
3834.0–60,541.1	68	---	---			
60,541.2–119,313.6	67	2.20	0.40, 12.01			
119,313.7–221,887.9	84	3.73	0.89, 15.64			
221,888.0–94,6875.0	73	1.90	0.46, 7.90			

Models were fit using a generalized linear model, with inverse-probability weighting and survey design-based standard errors. All models included maternal age at birth (<25, ≥25 & <35, ≥35 years) and insurance type used to pay for delivery of child (public, private, other). Quartiles are based on the distribution of controls. PFAS were modeled continuously in the mutually adjusted models (*n* = 86, *p*-value = 0.10); C_5_HF_9_O_3_ and C_13_H_2_F_24_O_2_ were excluded due to collinearity.

## Discussion

In this study of childhood ALL, we identified suggestive increased risks related to PFOA and PFOS in newborn DBS, and the risk was highest among the highest exposed children and those of non-Hispanic ethnicity. Our non-linear analyses of joint log2-PFOA and log2-PFOS showed that the highest risk of ALL was for children with the highest exposures when mutually adjusted for detection of other targeted PFAS. To our knowledge, this is the first study to examine the relationship between a comprehensive panel of PFAS assessed using non-targeted approaches in newborn DBS and ALL. While our analyses were limited by small sample sizes for participants with detectable levels, we did observe elevated significant risks for some non-targeted PFAS, emphasizing the need for consideration of PFAS beyond the commonly measured legacy and alternative chemicals. Future PFAS studies are encouraged to include untargeted approaches so that more data can be generated on these novel PFAS.

Our results for ALL and PFOS in DBS are similar to results in another California childhood ALL study [32] by Morimoto et al. (2025), who analyzed DBS from Childhood Cancer Record Linkage Project (CCRLP) for 122 ALL cases and 122 controls born throughout California during 2000–2008, while our study was restricted to LA County with births through 2015. Both studies showed elevated risks, although neither was statistically significant. In our analyses, the highest quartile of PFOS exposure was associated with a 56% increased risk of ALL and a doubling in continuous PFOS increased risk by 12%, compared to ORs of 1.30 and 1.04 for linear and branched PFOS in DBS, respectively [32]. While the strongest associations observed by Morimoto et al. were with MeFOSAA exposure detected in 64% of their DBS samples, we only detected the chemical in 4% of our DBS samples. This difference may be due to exposures declining over time or higher MeFOSAA detection limits in the current study. Temporal trends and analytic constraints may also explain the lower percentage of detection for PFOA and PFOS. Interestingly, Morimoto et al. did not detect PFOA in their DBS, although they did detect it in maternal blood samples.

Our results differ from two other childhood cancer studies in California (2000–2015) and Finland (1986-2010) that reported null associations with ALL and PFOS exposure [25, 33], although the Finnish study reported a positive association for pregnancy samples collected in 1986–1995, when PFOS levels were the highest [33]. These other studies examined exposure in maternal serum, and the California study modeled exposure based on steady-state drinking water exposure. The Finnish study observed elevated risks of ALL with PFOA exposure in pregnancy samples collected in 2001-2005 (OR = 1.56, 95% CI: 0.90, 2.69) and 2006-2010 (OR = 1.43, 95% CI:0.76, 2.68) and positive associations between ALL and MeFOSAA concentrations similar to Morimoto et al. [32].

Regarding PFAS identified by NTA approaches, we observed statistically significant positive associations for ALL with elevated C_4_HF_7_O_3_ and C_10_HF_19_O_5_ exposures. C_4_HF_7_O_3_ and C_10_HF_19_O_5_ are in a distinct class of PCFA-ethers that replaced PFOA in contemporary fluoropolymer manufacturing processes [34]. Exposures to these ether-linkage-containing PCFAs were notably higher among Asian/Pacific Islanders in our study population. We also observed higher levels of the H-substituted PFCAs identified by NTA approaches among Hispanic participants. Historically, legacy PFAS such as PFOA and PFOS have been consistently higher among non-Hispanic whites [35, 36] and those with higher socioeconomic status (SES) [36–38], which was also true for our participants. Varying exposure patterns by race/ethnicity are likely due to differences in diet and consumer product use. Very few studies have included NTA PFAS in biomonitoring [39–41], and none to our knowledge, have been able to examine differences by race/ethnicity or SES for comparison with our study results. Future studies that employ NTA approaches for PFAS biomonitoring should include a diverse population, if possible.

There are several strengths of this study. Dried blood spots were collected before cancer diagnosis and not subject to recall bias for exposure assessment. We were able to analyze samples for a large number of PFAS measured using hybrid approaches, making this one of the first studies to conduct NTA on DBS. We used a complex survey design, with inverse-probability weighting, to oversample exposed children based on whether their birth address was located within a public water district with detected PFOA or PFOS in UCMR3. We conducted linear and non-linear analyses of exposure with exploratory analyses by various strata. However, despite the efficient sampling design, the statistical power of our study was still limited by the small sample size and the fact that many of the 23 PFAS included in the targeted analysis were not detectable in most participants. Our analyses also relied on a single PFAS measure, which is arguably less reliable than repeated serum measures. As with other DBS studies, the smaller blood volumes and detection limits are also a constraint of this biomonitoring approach.

In conclusion, this is one of the first studies to measure targeted and non-targeted PFAS in dried blood spots and examine their relation to the risk of childhood ALL, an understudied health outcome. While not reaching statistical significance for most PFAS, the results are suggestive of a possible link between high levels of PFOA and PFOS exposure and ALL, particularly among non-Hispanic children. The results of the non-targeted PFAS analyses are less consistent but reveal possible associations that should be further investigated. Our findings support the contribution of newborn DBS as a media for assessing PFAS exposure, especially when examining childhood health outcomes.

## Supplementary information

Supplementary information

## Data Availability

The authors are not permitted to share data, as we are prohibited by California statutes from publicly sharing data that is derived from the California Department of Public Health. We welcome questions from other investigators or requests for additional analyses that are pertinent to the data presented in this manuscript. Other investigators can apply for the same data from the California Health and Human Services Agency Committee for the Protection of Human Subjects, as we have done for this study.
