# Supplementary material for: Targeted and non-targeted analyses of per-and polyfluoroalkyl substances in newborn dried blood spots and risk of childhood acute lymphoblastic leukemia
Source: J Expo Sci Environ Epidemiol. 2026 Apr 14;36(6):1183–93. doi: 10.1038/s41370-026-00891-6 (PMC13123734; doi:10.1038/s41370-026-00891-6)
Supplement: Supplementary file 1 [file 41370_2026_891_MOESM1_ESM.docx]

**Supplemental Information**

**Supplemental Figure S1. Correlations between Non-targeted PFAS Measures with >50% Detection Frequency.**

**Supplemental Table S1. Summary of Targeted PFAS Measured in Dried Blood Spots, Ordered from the Highest to the Lowest % Detection.**

**Supplemental Table S2. PFAS-like Molecular Features Identified by Non-Targeted Analysis in Dried Blood Spot Samples using Manual Integration or Peak Picking with FluoroMatch, Ordered from the Highest to the Lowest % Detection.**

**Supplemental Table S3: Feature parameters used in the Explorer software.**

**Supplemental Table S4. Summary of Imputed Targeted PFAS by Characteristics of Childhood Acute Lymphoblastic Leukemia (ALL) Participants in Los Angeles County, California (2000-2015)**

**Supplemental Table S5. Summary of Aromatic PFAS and Ether-linkage-containing PFCAs and PFSAs, Measured with Non-Targeted PFAS Approach, by Characteristics of Childhood Acute Lymphoblastic Leukemia (ALL) Participants in Los Angeles County, California (2000-2015)**

**Supplemental Table S6. Summary of H-substituted PFCAs, Measured with Non-Targeted PFAS Approach, by Characteristics of Childhood Acute Lymphoblastic Leukemia (ALL) Participants in Los Angeles County, California (2000-2015)**

**Supplemental Table S7. Ethnicity -Stratified Odds Ratios (ORs) and 95% Confidence Intervals (CI) for Associations between Targeted PFAS Measures in Dried Blood Spots and Childhood Acute Lymphoblastic Leukemia (ALL) in Los Angeles County, California (2000-2015).**

**Supplemental Table S8. Age at Diagnosis-Stratified Odds Ratios (ORs) and 95% Confidence Intervals (CI) for Associations between Targeted PFAS Measures in Dried Blood Spots and Childhood Acute Lymphoblastic Leukemia (ALL) in Los Angeles County, California (2000-2015).**

**Supplemental Table S9. Sex-Stratified Odds Ratios (ORs) and 95% Confidence Intervals (CI) for Associations between Targeted PFAS Measures in Dried Blood Spots and Childhood Acute Lymphoblastic Leukemia (ALL) in Los Angeles County, California (2000-2015).**

**Supplemental Table S10. Birth Order-Stratified Odds Ratios (ORs) and 95% Confidence Intervals (CI) for Associations between Targeted PFAS Measures in Dried Blood Spots and Childhood Acute Lymphoblastic Leukemia (ALL) in Los Angeles County, California (2000-2015).**

**Supplemental Table S11. Birthplace-Stratified Odds Ratios (ORs) and 95% Confidence Intervals (CI) for Associations between Targeted PFAS Measures in Dried Blood Spots and Childhood Acute Lymphoblastic Leukemia (ALL) in Los Angeles County, California (2000-2015).**

**Supplemental Table S12. Ethnicity-Stratified Odds Ratios (ORs) and 95% Confidence Intervals (CI) for Associations between Non-targeted PFAS Measures in Dried Blood Spots and Childhood Acute Lymphoblastic Leukemia (ALL) in Los Angeles County, California (2000-2015).**

**Supplemental Table S13. Age at Diagnosis-Stratified Odds Ratios (ORs) and 95% Confidence Intervals (CI) for Associations between Non-targeted PFAS Measures in Dried Blood Spots and Childhood Acute Lymphoblastic Leukemia (ALL) in Los Angeles County, California (2000-2015).**

**Supplemental Table S14. Sex-Stratified Odds Ratios (ORs) and 95% Confidence Intervals (CI) for Associations between Non-targeted PFAS Measures in Dried Blood Spots and Childhood Acute Lymphoblastic Leukemia (ALL) in Los Angeles County, California (2000-2015).**

**Supplemental Table S15. Birth Order-Stratified Odds Ratios (ORs) and 95% Confidence Intervals (CI) for Associations between Non-targeted PFAS Measures in Dried Blood Spots and Childhood Acute Lymphoblastic Leukemia (ALL) in Los Angeles County, California (2000-2015).**

**Supplemental Table S16. Birthplace-Stratified Odds Ratios (ORs) and 95% Confidence Intervals (CI) for Associations between Non-targeted PFAS Measures in Dried Blood Spots and Childhood Acute Lymphoblastic Leukemia (ALL) in Los Angeles County, California (2000-2015).**

**Supplemental Figure S1. Correlations between Non-targeted PFAS Measures with >50% Detection Frequency.** Spearman correlation coefficients are reported. Correlations greater than 0.85 (in bold) were considered highly correlated.

|  | C4HF7O3 | C4HF9O4S | C5HF9O3 | C9H2F16O2 | C14H2F26O2 | C15H2F28O2 | C13H2F24O2 | C10HF19O5 |
| --- | --- | --- | --- | --- | --- | --- | --- | --- |
| C4HF7O3 | 1.00 |  |  |  |  |  |  |  |
| C4HF9O4S | 0.65 | 1.00 |  |  |  |  |  |  |
| C5HF9O3 | **0.88** | 0.76 | 1.00 |  |  |  |  |  |
| C9H2F16O2 | 0.48 | 0.49 | 0.54 | 1.00 |  |  |  |  |
| C14H2F26O2 | 0.27 | 0.05 | 0.11 | -0.02 | 1.00 |  |  |  |
| C15H2F28O2 | 0.12 | -0.03 | -0.08 | -0.05 | 0.82 | 1.00 |  |  |
| C13H2F24O2 | 0.23 | 0.00 | 0.04 | -0.07 | **0.87** | 0.80 | 1.00 |  |
| C10HF19O5 | 0.48 | 0.07 | 0.36 | 0.23 | 0.31 | 0.29 | 0.29 | 1.00 |
| C16H8Cl2F6N2O2S | 0.49 | 0.55 | 0.60 | 0.44 | -0.22 | -0.34 | -0.28 | 0.08 |

| **Supplemental Table S1. Summary of Targeted PFAS Measured in Dried Blood Spots, Ordered from the Highest to the Lowest % Detection.** | | | | | | | | |
| --- | --- | --- | --- | --- | --- | --- | --- | --- |
| **Compound ^1^** | **Abbreviation**  **and Class** | | **LOD (pg/µL)** | | **% Detect** | **Concentration (pg/g dried blood) ^2^** | | |
|  |  |  | **Card** | **Blank** | **N=344** | **Mean** | **SD** | **Range** |
| Perfluoro-*n*-octanoic acid | PFOA | PFCA | 0.1165 | 0.1473 | 62% | 6111.1 | 5886.7 | (181.9, 61855.3) |
| Perfluoro-1-octanesulfonate | PFOS | PFSA | 0.1336 | 0.1009 | 57% | 12200.2 | 11631.7 | (185.5, 83893.0) |
| Perfluoro-1-hexanesulfonate | PFHxS | PFSA | 0.0357 | 0.0383 | 40% | 3354.3 | 6973.8 | (168.2, 69197.5) |
| Perfluorooctane sulfonamide | FOSA | FOSA | 0.0283 | 0.0247 | 26% | 3571.6 | 3854.5 | (140.8, 22242.6) |
| Perfluoro-n-pentanoic acid | PFPeA | PFCA | 0.0596 | 0.0230 | 24% | 44977.0 | 101970.7 | (290.1, 700577.9) |
| 1H,1H,2H,2H-Perfluoro-1-octanesulfonate | 6:2 FTS | PreFAS | 0.1000 | 0.1000 | 17% | 5042.7 | 4721.0 | (108.4, 26319.4) |
| Perfluoro-n-butanoic acid | PFBA | PFCA | 0.0546 | 0.0551 | 9% | 2121.1 | 2799.0 | (60.6, 12552.6) |
| N-ethylperfluoro-1-octanesulfonamide | N-EtFOSA-M | PreFAS | 0.0500 | 0.0455 | 8% | 1856.1 | 1767.5 | (144.8, 6898.4) |
| Perfluoro-*n*-hexanoic acid | PFHxA | PFCA | 0.0482 | 0.0611 | 8% | 4495.6 | 4952.9 | (41.5, 19143.7) |
| Perfluoro-1-butanesulfonate | PFBS | PFSA | 0.0561 | 0.0502 | 6% | 3759.7 | 6795.2 | (32.4, 26078.7) |
| 1H,1H,2H,2H-Perfluorodecane sulfonate | 8:2 FTS | PreFAS | 0.0488 | 0.0338 | 4% | 2562.9 | 3844.8 | (534.1, 14927.8) |
| N-methylperflouro-1-octanesulfonamide | N-MeFOSA-M | PreFAS | 0.0689 | 0.1027 | 4% | 6309.4 | 14307.8 | (1066.1, 53662.5) |
| Perfluoro-1-decanesulfonate | PFDS | PFSA | 0.0217 | 0.0204 | 3% | 2184.3 | 3542.4 | (256.4, 11960.9) |
| Perfluoro-*n*-dodecanoic acid | PFDoA | PFCA | 0.0708 | 0.0627 | 3% | 5047.8 | 3593.3 | (1533.1, 12293.4) |
| Perfluoro-*n*-decanoic acid | PFDA | PFCA | 0.0387 | 0.0322 | 2% | 2052.8 | 2803.0 | (188.1, 8753.0) |
| Perfluoro-*n*-nonanoic acid | PFNA | PFCA | 0.0402 | 0.0646 | 2% | 3104.9 | 3228.0 | (1391.2, 9942.7) |
| Perfluoro-1-heptanesulfonate | PFHpS | PFSA | 0.0700 | 0.1593 | 1% | 4576.9 | 2765.7 | (2007.4, 8485.4) |
| Perfluoro-*n*-tridecanoic acid | PFTrDA | PFCA | 0.1513 | 0.1550 | 0% | NA | NA | NA |
| 1H,1H,2H,2H-perfluorohexane sulfonate | 4:2 FTS | PreFAS | 0.2767 | 0.1181 | 0% | NA | NA | NA |
| Perfluoro-1-nonanesulfonate | PFNS | PFSA | 0.0258 | 0.0241 | 0% | NA | NA | NA |
| Perfluoro-1-pentanesulfonate | PFPeS | PFSA | 0.0358 | 0.0482 | 0% | NA | NA | NA |
| Perfluoro-*n*-tetradecanoic acid | PFTeDA | PFCA | 0.1000 | 0.1000 | 0% | NA | NA | NA |
| Perfluoro-*n*-undecanoic acid | PFUnA | PFCA | 0.0810 | 0.0375 | 0% | NA | NA | NA |

^1^ Perfluoro‐*n*‐heptanoic acid (PFHpA) might have been introduced into the samples during sample collection, transport or storage before the PFAS analysis so was excluded from statistical analyses.

^2^ Summary statistics are for non-imputed data

**Supplemental Table S2. PFAS-like Molecular Features Identified by Non-Targeted Analysis in Dried Blood Spot Samples using Manual Integration or Peak Picking with FluoroMatch, Ordered from the Highest to the Lowest % Detection.**

| **Class** | **Possible Name** | | **Formula** | | **Detection Frequency** | | **Internal Standard** | ***m/z*** | **Retention Time (min)** |
| --- | --- | --- | --- | --- | --- | --- | --- | --- | --- |
| **Manual Integration of Semi-quantitative PFAS** | | | | | | |  |  |  |
| PFCA-ether | | Perfluoro-3-methoxypropanoic acid | | C4HF7O3 | | 85% | M5PFPeA | 228.9744 | 2.163 |
| PFSA-ether | | Perfluoro(2-ethoxyethane)sulfonic acid | | C4HF9O4S | | 84% | M5PFPeA | 314.9383 | 3.231 |
| PFCA-ether | | Difluoro(perfluoropropoxy)acetic acid | | C5HF9O3 | | 78% | M5PFPeA | 228.9744 | 2.163 |
| PFCA-H | | H-Perfluorononanoic acid | | C9H2F16O2 | | 78% | M9PFNA | 444.9730 | 3.623 |
| PFCA-H | | H-Perfluorotetradecanoic acid | | C14H2F26O2 | | 60% | M6PFDA | 694.9568 | 4.537 |
| PFCA-H | | H-Perfluoropentadecanoic acid | | C15H2F28O2 | | 57% | M6PFDA | 744.9540 | 4.718 |
| PFCA-H | | H-Perfluorotridecanoic acid | | C13H2F24O2 | | 50% | M6PFDA | 644.9605 | 4.284 |
| PFCA-ether | | Perfluoro-3,6,9-trioxatridecanoic acid | | C10HF19O5 | | 50% | M6PFDA | 560.9448 | 4.263 |
| PFCA-H | | H-Perfluoroheptadecanoic acid | | C17H2F32O2 | | 48% | M6PFDA | 844.9478 | 5.235 |
| PFCA-H | | H-Perfluorohenicosanoic acid | | C21H2F40O2 | | 41% | M6PFDA | 1044.9360 | 6.395 |
| PFCA-H | | H-Perfluorooctadecanoic acid | | C18H2F34O2 | | 36% | M6PFDA | 894.9441 | 5.589 |
| PFCA-H | | H-Perfluorododecanoic acid | | C12H2F22O2 | | 29% | M6PFDA | 594.9635 | 4.096 |
| PFCA-H | | H-Perfluoroeicosanoic acid | | C20H2F38O2 | | 29% | M6PFDA | 994.9379 | 6.157 |
| PFCA-H | | H-Perfluorononadecanoic acid | | C19H2F36O2 | | 28% | M6PFDA | 944.9395 | 5.804 |
| PFCA-H | | H-Perfluorodecanoic acid | | C10H2F18O2 | | 24% | M6PFDA | 494.9698 | 3.783 |
| PFCA-H | | H-Perfluorodocosanoic acid | | C22H2F42O2 | | 21% | M6PFDA | 1094.932 | 6.678 |
| PFSA-ether | | Perfluoro diethernonane sulfonic acid | | C7HF15O5S | | 18% | M4PFHpA | 480.9232 | 3.877 |
| PFCA-H | | H-Perfluoroundecanoic acid | | C11H2F20O2 | | 13% | M6PFDA | 544.9664 | 3.935 |
| **Peak Picking of Other NTA PFAS** | | | | | | |  |  |  |
| Aromatic PFAS | N-((2,5-Dichloro-4-(1,1,2,2-tetrafluoroethylsulfanyl)phenyl)carbamoyl)-2,6-difluorobenzamide | | C16H8Cl2F6N2O2S | | 85% | |  | 474.9530 | 3.533 |
| Unknown | Feature 828 | | Unknown | | 42% | |  | 244.9080 | 3.213 |
| Unknown | Feature 1598 | | Unknown | | 26% | |  | 466.9585 | 4.266 |
| Unknown | Feature 9170 | | Unknown | | 22% | |  | 168.9992 | 1.198 |
| Unknown | Feature 5217 | | Unknown | | 20% | |  | 518.9805 | 4.138 |
| Unknown | Feature 228 | | Unknown | | 8% | |  | 165.9792 | 2.933 |
| Unknown | Feature 8971 | | Unknown | | 7% | |  | 714.9534 | 5.088 |
| Unknown | Feature 3027 | | Unknown | | 5% | |  | 290.9910 | 3.137 |

**Supplemental Table S3: Feature parameters used in the Explorer software.**

| **Ion Species** |  |
| --- | --- |
| Positive Ions | NA |
| Negative Ions | -H, +Cl, +CH3COO |
| Neutral Modifications | -F, -HF |
| Isotope Model | Unbiased |
| Expected Data Charge State | 1 to 2 |
| Use Multi-pass Exhaustive Grouping | Unchecked |
| Filter by Mass Defect | Unchecked |
| Cross-Sample Variation Max. Time Shift | +/- (0.3 min+0%) |
| **Compound Alignment** |  |
| RT Tolerance | +/- 0.1 min |
| Mass Tolerance | +/- 100 ppm |
| Score (MFE) | >= 70 |
| Filter Conditions Must Apply In At Least | 1 file, across all sample files |
| **Gap Filling** |  |
| Possible *m/z* | Symmetric (ppm), +/- 35 |
| RT Extraction Factor | 3 |
| Baseline to Zero | Checked |
| Smooth EIC Before Integration | Checked |
| Function | Gaussian |
| Function Width | 5 pts |
| Gaussian Width | 3 pts |
| **Spectrum Extraction** |  |
| Mass Spectra to Include | Average scans >10% of peak height |
| TOF Spectra | Exclude if above 20% of saturation |
| Never Return an Empty Spectrum | Checked |
| **Score Weighting** |  |
| Mass Score | Weight 100, Expected variation 5.6 ppm |
| Isotope Abundance | Weight 60, Expected variation 7.5% |
| Isotope Spacing Score | Weight 50 |
| Retention Time Score | Weight 100, Expected variation 0.115 min |
| **Results Filters and Warnings** |  |
| Low Score Matches | Warn if score is <75 |
| Single Ion Matches | None selected |

| **Supplemental Table S4. Summary of Imputed Targeted PFAS by Characteristics of Childhood Acute Lymphoblastic Leukemia (ALL) Participants in Los Angeles County, California (2000-2015).** P-values are from design-based ANOVA. | | | | | |
| --- | --- | --- | --- | --- | --- |
|  | **n** | **PFOA** | | **PFOS** | |
|  |  | Mean (SD) | P (χ^2^) | Mean (SD) | P (χ^2^) |
| **Child’s birth year** |  |  | <0.01 |  | <0.01 |
| 2000-2004 | 134 | 6000.6 (4447.4) |  | 15492.0 (11388.4) |  |
| 2005-2009 | 115 | 3976.0 (3842.3) |  | 6182.4 (5501.4) |  |
| 2010-2015 | 95 | 2679.1 (3212.8) |  | 3054.1 (3887.9) |  |
| **Child’s sex** |  |  | 0.59 |  | 0.79 |
| Male | 166 | 4605.9 (4721.4) |  | 8919.2 (9556.4) |  |
| Female | 178 | 4225.1 (3574.4) |  | 8943.6 (9774.5) |  |
| **Child’s race/ethnicity** |  |  | 0.49 |  | 0.13 |
| Non-Hispanic White | 66 | 5475.2 (4669.2) |  | 11596.9 (11246.9) |  |
| Non-Hispanic Black | 7 | 2878.0 (1805.9) |  | 8267.3 (6284.3) |  |
| Hispanic | 244 | 4220.1 (3991.5) |  | 8439.8 (9466.5) |  |
| Asian/Pacific Islander | 24 | 4122.8 (4621.1) |  | 7576.0 (6825.3) |  |
| Other or unknown | 3 | 1639.0 (916.4) |  | 5230.0 (5994.5) |  |
| **Birth order** |  |  | 0.53 |  | 0.59 |
| First child | 141 | 4973.0 (4609.2) |  | 9677.8 (11064.1) |  |
| Second child | 111 | 3893.9 (3836.9) |  | 7932.6 (8156.3) |  |
| Third or more | 92 | 4132.7 (3692.1) |  | 9000.3 (8989.9) |  |
| **Maternal age (years)** |  |  | 0.81 |  | 0.12 |
| < 25 | 108 | 4077.3 (3881.0) |  | 6775.3 (6882.1) |  |
| ≥ 25 & < 35 | 172 | 4619.7 (4094.1) |  | 9664.0 (10032.5) |  |
| ≥ 35 | 64 | 4394.9 (4758.2) |  | 10587.1 (11919.4) |  |
| **Paternal age (years)** |  |  | 0.19 |  | 0.02 |
| < 25 | 64 | 4160.0 (3745.3) |  | 6814.9 (6968.0) |  |
| ≥ 25 & < 35 | 148 | 4517.1 (4117.8) |  | 8492.7 (8057.7) |  |
| ≥ 35 | 132 | 4400.6 (4403.2) |  | 10454.3 (11976.8) |  |
| **Maternal education** |  |  | 0.26 |  | 0.01 |
| High school or less | 182 | 4173.3 (3472.2) |  | 8119.4 (8080.7) |  |
| Some college or more | 156 | 4705.5 (4872.1) |  | 9883.5 (11231.7) |  |
| Missing | 6 | 3673.0 (2733.6) |  | 9172.2 (9152.9) |  |
| **Paternal education** |  |  | 0.26 |  | < 0.01 |
| High school or less | 170 | 4132.9 (3823.3) |  | 7928.8 (8407.3) |  |
| Some college or more | 138 | 5075.6 (4776.0) |  | 10789.0 (11275.7) |  |
| Missing | 36 | 3137.4 (2390.6) |  | 6676.5 (7320.5) |  |
| **Insurance for delivery** |  |  | 0.20 |  | < 0.01 |
| Private | 178 | 5193.7 (4343.0) |  | 12048.4 (11285.7) |  |
| Public | 152 | 3449.9 (3608.6) |  | 5608.3 (6101.1) |  |
| Other | 14 | 4525.5 (5244.6) |  | 5822.0 (5242.3) |  |
| **Mother’s country of birth** |  |  | 0.64 |  | 0.02 |
| Mexico | 69 | 3842.7 (3777.9) |  | 7235.4 (7483.8) |  |
| US 50 states | 212 | 4629.5 (4266.9) |  | 9680.6 (10670.4) |  |
| Other or unknown | 63 | 4246.1 (4163.0) |  | 8278.9 (7861.0) |  |
| **Median house value ($1000)** |  |  | <0.01 |  | <0.01 |
| ≥ 10.0 & < 142.8 | 79 | 4149.5 (4311.4) |  | 8027.3 (10708.8) |  |
| ≥ 142.8 & < 162.6 | 73 | 3956.3 (3340.8) |  | 7990.0 (8394.8) |  |
| ≥ 162.6 & < 197.7 | 93 | 4363.6 (3775.0) |  | 9002.3 (9412.7) |  |
| ≥ 197.7 & ≤ 1000.0 | 92 | 4990.0 (4988.7) |  | 10527.9 (10107.9) |  |
| Missing | 7 | 4963.0 (3006.6) |  | 7020.1 (3756.1) |  |
| **Water district** |  |  | 0.48 |  | 0.08 |
| Unexposed water district | 172 | 4600.8 (4750.4) |  | 8808.7 (10890.1) |  |
| CA Water Service East Los Angeles | 25 | 3461.8 (3492.4) |  | 6009.3 (7166.7) |  |
| City of Downey Water Department | 22 | 4684.5 (3744.1) |  | 9611.4 (8530.0) |  |
| Golden State Water Co - Norwalk | 18 | 4060.1 (3199.9) |  | 9896.8 (10420.3) |  |
| Montebello Land & Water Co | 15 | 3549.7 (2141.0) |  | 6470.2 (4114.1) |  |
| Orchard Dale Water District | 6 | 4867.2 (1399.3) |  | 10737.2 (6301.2) |  |
| Park Water Co - Bellflower/Norwalk | 14 | 5533.3 (3387.2) |  | 10273.8 (9853.7) |  |
| City of Pico Rivera Water Department | 11 | 3574.5 (3488.5) |  | 10215.7 (8815.1) |  |
| Pico Water District | 13 | 4384.4 (5471.7) |  | 10190.8 (7932.1) |  |
| Santa Clarita Water Division | 20 | 4162.9 (3348.5) |  | 11074.0 (10618.3) |  |
| Valencia Water Company | 16 | 4768.3 (3048.0) |  | 11047.6 (5747.3) |  |

| **Supplemental Table S5. Summary of Aromatic PFAS and Ether-linkage-containing PFCAs and PFSAs, Measured with Non-Targeted PFAS Approach, by Characteristics of Childhood Acute Lymphoblastic Leukemia (ALL) Participants in Los Angeles County, California (2000-2015).** Semi-quantitative non-targeted PFAS analyses measure ng chemical/ g dried blood. Non-targeted analyses of C_16_H_8_C_l2_F_6_N_2_O_2_S measure normalized peak area of chemical. P-values are from design-based ANOVA. | | | | | | | | | | |
| --- | --- | --- | --- | --- | --- | --- | --- | --- | --- | --- |
|  | **C_16_H_8_Cl_2_F_6_N_2_O_2_S** | | **C_4_HF_7_O_3_** | | **C_4_HF_9_O_4_S** | | **C_5_HF_9_O_3_** | | **C_10_HF_19_O_5_** | |
|  | Mean (SD) | P (χ^2^) | Mean (SD) | P (χ^2^) | Mean (SD) | P (χ^2^) | Mean (SD) | P (χ^2^) | Mean (SD) | P (χ^2^) |
| **Child’s birth year** |  | 0.03 |  | 0.76 |  | 0.77 |  | 0.27 |  | 0.23 |
| 2000-2004 | 182156.7 (159720) |  | 29.6 (20.2) |  | 153.1 (171.0) |  | 13.5 (10.0) |  | 12.9 (12.9) |  |
| 2005-2009 | 158856.3 (127912.1) |  | 38.6 (53.6) |  | 167.0 (364.7) |  | 17.5 (21.7) |  | 31.4 (67.2) |  |
| 2010-2015 | 144242.8 (117549.1) |  | 34.1 (30.6) |  | 178.0 (196.0) |  | 17.2 (16.2) |  | 23.7 (47.0) |  |
| **Child’s sex** |  | 0.72 |  | 0.12 |  | 0.03 |  | 0.06 |  | 0.28 |
| Male | 162937.8 (120721.5) |  | 39.1 (49.1) |  | 182.4 (331.1) |  | 18.3 (21.5) |  | 28.6 (64.7) |  |
| Female | 165607.9 (155603.9) |  | 29.0 (20.4) |  | 146.7 (152.5) |  | 13.8 (9.7) |  | 15.7 (16.3) |  |
| **Child’s race/ethnicity** |  | 0.05 |  | 0.10 |  | 0.80 |  | 0.16 |  | 0.35 |
| Non-Hispanic White | 175918.3 (160668.3) |  | 39.2 (28.8) |  | 179.9 (175.2) |  | 18.0 (17.0) |  | 28.9 (53.1) |  |
| Non-Hispanic Black | 196311.4 (140279.6) |  | 29.4 (16.0) |  | 131.2 (74.9) |  | 15.1 (9.0) |  | 7.6 (0.6) |  |
| Hispanic | 154052.7 (132962.9) |  | 29.7 (24.6) |  | 146.3 (166.0) |  | 14.1 (11.1) |  | 15.7 (17.3) |  |
| Asian/Pacific Islander | 214274.9 (144534.8) |  | 60.0 (101.5) |  | 291.0 (716.0) |  | 24.8 (39.2) |  | 51.7 (121.1) |  |
| Other or unknown | 232234.1 (89799.8) |  | 50.1 (17.2) |  | 184.2 (32.1) |  | 24.3 (7.6) |  | 58.1 (3.6) |  |
| **Birth order** |  | 0.59 |  | 0.07 |  | 0.23 |  | 0.28 |  | 0.26 |
| First child | 170932.3 (146275) |  | 35.8 (48.5) |  | 185.0 (353.4) |  | 17.4 (20.9) |  | 24.6 (60.3) |  |
| Second child | 162828.3 (129424.6) |  | 31.7 (28.6) |  | 157.6 (188.5) |  | 14.4 (14.3) |  | 19.8 (44.2) |  |
| Third or more | 156394.8 (142206.8) |  | 33.2 (24.8) |  | 141.4 (117.9) |  | 15.5 (10.8) |  | 20.3 (20.4) |  |
| **Maternal age (years)** |  | 0.38 |  | 0.18 |  | 0.27 |  | 0.24 |  | 0.37 |
| < 25 | 161501.7 (126806) |  | 29.3 (20.1) |  | 159.8 (177.6) |  | 15.3 (10.3) |  | 17.0 (20.7) |  |
| ≥ 25 & < 35 | 154717.1 (127467.7) |  | 32.4 (26.3) |  | 151.8 (165.6) |  | 14.4 (11.7) |  | 17.5 (16.2) |  |
| ≥ 35 | 194096.7 (182948.3) |  | 45.8 (70.2) |  | 203.6 (469.8) |  | 21.0 (29.7) |  | 42.1 (100.1) |  |
| **Paternal age (years)** |  | <0.01 |  | 0.07 |  | 0.64 |  | 0.06 |  | 0.18 |
| < 25 | 166417.5 (148155.9) |  | 30.3 (21.1) |  | 173.9 (207.4) |  | 15.5 (11.0) |  | 15.9 (17.0) |  |
| ≥ 25 & < 35 | 157933.3 (141753.3) |  | 31.2 (24.3) |  | 143.3 (140.9) |  | 14.8 (12.0) |  | 18.2 (19.6) |  |
| ≥ 35 | 170490 (133826.8) |  | 38.5 (52.3) |  | 183.6 (356.9) |  | 17.5 (22.3) |  | 29.8 (73.7) |  |
| **Maternal education** |  | 0.05 |  | 0.06 |  | 0.43 |  | 0.04 |  | 0.27 |
| High school or less | 160058.7 (140183.6) |  | 30.0 (23.2) |  | 150.5 (173.8) |  | 14.1 (10.1) |  | 15.6 (17.5) |  |
| Some college or more | 163971.9 (137346.7) |  | 37.3 (49.1) |  | 173.3 (325.2) |  | 17.2 (20.8) |  | 28.3 (65.3) |  |
| Missing | 281280.9 (148294.3) |  | 59.7 (37.4) |  | 298.6 (187.6) |  | 31.2 (27.6) |  | 33.6 (29.1) |  |
| **Paternal education** |  | <0.01 |  | <0.01 |  | 0.23 |  | <0.01 |  | 0.24 |
| High school or less | 165212.8 (141918.3) |  | 29.7 (24.2) |  | 153.1 (165.4) |  | 14.0 (10.0) |  | 16.3 (18.0) |  |
| Some college or more | 159036.2 (115529.4) |  | 38.7 (50.4) |  | 175.6 (345.9) |  | 17.2 (21.3) |  | 30.0 (70.8) |  |
| Missing | 181421.1 (206706) |  | 34.9 (27.8) |  | 169.1 (143.8) |  | 20.2 (17.9) |  | 18.9 (19.3) |  |
| **Insurance for delivery** |  | 0.38 |  | 0.03 |  | 0.21 |  | 0.29 |  | 0.47 |
| Private | 173436.9 (146030.8) |  | 36.7 (45.6) |  | 183.2 (321.9) |  | 17.3 (20.0) |  | 25.4 (62.1) |  |
| Public | 150069.5 (124535.5) |  | 30.5 (26.6) |  | 142.3 (155.8) |  | 13.9 (11.2) |  | 18.1 (19.6) |  |
| Other | 202756.1 (198249.3) |  | 35.4 (23.7) |  | 148.8 (96.8) |  | 18.7 (14.6) |  | 19.4 (14.0) |  |
| **Mother’s country of birth** |  | 0.89 |  | 0.35 |  | 0.43 |  | 0.31 |  | 0.18 |
| Mexico | 169255.8 (168888) |  | 32.0 (32.8) |  | 166.1 (212.5) |  | 13.9 (13.2) |  | 16.3 (20.8) |  |
| US 50 states | 164749.5 (134657.8) |  | 31.3 (20.5) |  | 152.9 (147.8) |  | 15.3 (11.4) |  | 16.5 (16.3) |  |
| Other or unknown | 157476.9 (123814) |  | 44.9 (72.2) |  | 200.8 (492.2) |  | 20.6 (30.1) |  | 46.4 (101.0) |  |
| **Median house value ($1000)** |  | 0.22 |  | 0.12 |  | <0.01 |  | 0.86 |  | 0.17 |
| ≥ 10.0 & < 142.8 | 164585.1 (147897.6) |  | 35.6 (60.0) |  | 205.7 (445.1) |  | 18.2 (25.7) |  | 31.9 (85.9) |  |
| ≥ 142.8 & < 162.6 | 164395.8 (155670.5) |  | 32.3 (25.7) |  | 157.0 (175.1) |  | 15.1 (10.5) |  | 14.5 (12.6) |  |
| ≥ 162.6 & < 197.7 | 159970.3 (124185.2) |  | 31.5 (27.4) |  | 142.0 (162.2) |  | 14.5 (13.1) |  | 15.8 (16.9) |  |
| ≥ 197.7 & ≤ 1000.0 | 169159.3 (138487.4) |  | 35.7 (28.8) |  | 157.1 (165.5) |  | 16.0 (14.2) |  | 26.7 (44.8) |  |
| Missing | 153951.1 (94416.24) |  | 35.8 (21.6) |  | 169.8 (128.0) |  | 15.1 (10.0) |  | 21.2 (25.4) |  |

| **Supplemental Table S6. Summary of H-substituted PFCAs, Measured with Non-Targeted PFAS Approach, by Characteristics of Childhood Acute Lymphoblastic Leukemia (ALL) Participants in Los Angeles County, California (2000-2015).** Semi-quantitative non-targeted PFAS analyses measure ng chemical/ g dried blood. P-values are from design-based ANOVA. | | | | | | | | |
| --- | --- | --- | --- | --- | --- | --- | --- | --- |
|  | **C_9_H_2_F_16_O_2_** | | **C_14_H_2_F_26_O_2_** | | **C_15_H_2_F_28_O_2_** | | **C_13_H_2_F_24_O_2_** | |
|  | Mean (SD) | P (χ^2^) | Mean (SD) | P (χ^2^) | Mean (SD) | P (χ^2^) | Mean (SD) | P (χ^2^) |
| **Child’s birth year** |  | 0.66 |  | 0.07 |  | 0.04 |  | 0.07 |
| 2000-2004 | 31.8 (26.7) |  | 38.4 (24.7) |  | 31.2 (20.0) |  | 39.9 (17.9) |  |
| 2005-2009 | 31.1 (44.9) |  | 49.3 (27.0) |  | 37.5 (18.8) |  | 53.6 (27.1) |  |
| 2010-2015 | 27.8 (22.0) |  | 51.6 (43.2) |  | 39.0 (25.4) |  | 58.0 (36.9) |  |
| **Child’s sex** |  | 0.07 |  | 0.23 |  | 0.10 |  | 0.61 |
| Male | 33.9 (42.6) |  | 47.0 (29.6) |  | 36.4 (21.1) |  | 48.9 (27.4) |  |
| Female | 27.1 (19.8) |  | 44.4 (34.7) |  | 34.5 (21.9) |  | 50.4 (29.6) |  |
| **Child’s race/ethnicity** |  | 0.77 |  | 0.17 |  | 0.04 |  | 0.23 |
| White | 31.0 (22.5) |  | 40.4 (25.8) |  | 30.7 (15.5) |  | 43.1 (20.8) |  |
| Black | 27.0 (20.6) |  | 37.4 (18.7) |  | 23.3 (6.8) |  | 36.9 (8.1) |  |
| Hispanic | 28.5 (23.3) |  | 48.1 (35.0) |  | 37.4 (23.3) |  | 51.9 (30.7) |  |
| Asian/Pacific Islander | 46.8 (86.3) |  | 40.4 (22.1) |  | 30.1 (16.9) |  | 47.0 (25.5) |  |
| Other or unknown | 33.2 (13.0) |  | 44.7 (17.7) |  | 36.8 (13.4) |  | 47.7 (14.8) |  |
| **Birth order** |  | 0.04 |  | 0.25 |  | 0.55 |  | 0.24 |
| First child | 35.5 (47.0) |  | 48.4 (36.8) |  | 35.6 (21.3) |  | 54.8 (33.1) |  |
| Second child | 25.8 (19.5) |  | 49.4 (33.3) |  | 38.8 (25.8) |  | 51.2 (28.9) |  |
| Third or more | 28.5 (16.2) |  | 36.6 (18.2) |  | 31.0 (14.4) |  | 39.5 (14.4) |  |
| **Maternal age (years)** |  | 0.86 |  | 0.73 |  | 0.81 |  | 0.39 |
| < 25 | 30.4 (27.5) |  | 49.3 (41.3) |  | 37.4 (26.1) |  | 52.3 (36.7) |  |
| ≥ 25 & < 35 | 28.2 (20.0) |  | 47.4 (28.0) |  | 36.2 (19.7) |  | 50.8 (25.9) |  |
| ≥ 35 | 36.4 (58.5) |  | 36.7 (24.2) |  | 30.5 (17.1) |  | 42.7 (17.4) |  |
| **Paternal age (years)** |  | 0.48 |  | 0.05 |  | 0.04 |  | 0.30 |
| < 25 | 30.1 (30.5) |  | 50.1 (35.3) |  | 40.4 (25.6) |  | 57.0 (35.8) |  |
| ≥ 25 & < 35 | 28.7 (19.3) |  | 46.8 (27.4) |  | 36.0 (20.2) |  | 49.7 (26.1) |  |
| ≥ 35 | 32.5 (44.5) |  | 42.0 (35.7) |  | 31.9 (20.1) |  | 45.4 (26.2) |  |
| **Maternal education** |  | 0.49 |  | 0.34 |  | 0.49 |  | 0.26 |
| High school or less | 27.5 (17.5) |  | 47.7 (35.9) |  | 36.2 (21.5) |  | 50.6 (30.9) |  |
| Some college or more | 33.0 (43.7) |  | 43.5 (27.9) |  | 34.5 (22.0) |  | 48.5 (26.0) |  |
| Missing | 49.6 (48.8) |  | 48.2 (16.1) |  | 33.4 (15.1) |  | 48.7 (12.3) |  |
| **Paternal education** |  | 0.31 |  | 0.50 |  | 0.56 |  | 0.42 |
| High school or less | 27.3 (20.0) |  | 45.5 (29.4) |  | 36.8 (21.7) |  | 49.0 (26.7) |  |
| Some college or more | 33.2 (44.3) |  | 45.0 (28.4) |  | 33.6 (19.8) |  | 50.9 (27.0) |  |
| Missing | 33.3 (27.8) |  | 49.7 (51.9) |  | 36.7 (26.7) |  | 47.6 (38.5) |  |
| **Insurance for delivery** |  | 0.11 |  | 0.37 |  | 0.22 |  | 0.69 |
| Private | 34.4 (42.1) |  | 46.8 (35.6) |  | 36.3 (22.4) |  | 50.0 (29.4) |  |
| Public | 26.0 (17.9) |  | 46.0 (28.8) |  | 35.6 (21.1) |  | 49.8 (27.9) |  |
| Other | 27.0 (16.1) |  | 32.9 (14.7) |  | 25.6 (12.9) |  | 45.9 (26.0) |  |
| **Mother’s country of birth** |  | 0.66 |  | 0.16 |  | 0.12 |  | 0.44 |
| Mexico | 29.3 (23.9) |  | 42.7 (27.4) |  | 32.3 (19.7) |  | 41.2 (19.1) |  |
| US 50 states | 29.4 (23.8) |  | 48.3 (36.1) |  | 37.6 (23.4) |  | 53.4 (32.5) |  |
| Other or unknown | 34.9 (57.9) |  | 40.5 (19.8) |  | 31.9 (15.4) |  | 47.2 (19.9) |  |
| **Median house value ($1000)** |  | 0.39 |  | 0.15 |  | 0.12 |  | 0.06 |
| ≥ 10.0 & < 142.8 | 38.7 (56.5) |  | 41.6 (27.0) |  | 36.4 (22.0) |  | 44.0 (20.2) |  |
| ≥ 142.8 & < 162.6 | 27.3 (20.6) |  | 46.7 (31.0) |  | 37.0 (25.7) |  | 50.3 (31.3) |  |
| ≥ 162.6 & < 197.7 | 29.8 (24.4) |  | 48.3 (40.9) |  | 34.3 (21.6) |  | 52.1 (35.2) |  |
| ≥ 197.7 & ≤ 1000.0 | 26.4 (16.8) |  | 44.6 (25.9) |  | 34.0 (18.1) |  | 49.8 (24.0) |  |
| Missing | 26.8 (14.3) |  | 79.5 (62.2) |  | 41.3 (22.3) |  | 89.4 (43.3) |  |
|  | |  |  |  |  |  |  |  |

| **Supplemental Table S7. Ethnicity-Stratified Odds Ratios (ORs) and 95% Confidence Intervals (CI) for Associations between Targeted PFAS Measures in Dried Blood Spots and Childhood Acute Lymphoblastic Leukemia (ALL) in Los Angeles County, California (2000-2015)** | | | | | | |
| --- | --- | --- | --- | --- | --- | --- |
|  | **Hispanic** | | | **Non-Hispanic** | | |
| **Chemical (pg/g dried blood)^1^** | **n cases/ controls** | **OR** | **95% CI** | **n cases/ controls** | **OR** | **95% CI** |
| **Continuous** | | | | | | |
| Log2 PFOA | 89/148 | 1.01 | 0.72, 1.42 | 32/66 | 1.76 | 1.02, 3.02 |
| Log2 PFOS | 91/150 | 0.95 | 0.68, 1.32 | 33/66 | 1.66 | 1.03, 2.66 |
| **Categorical ^2^** | | | | | | |
| PFOA |  |  |  |  |  |  |
| 2.14 – 1365.89 | 22/41 | --- | --- | 3/13 | --- | --- |
| 1365.89 – 3136.46 | 20/35 | 1.03 | 0.22, 4.87 | 9/18 | 4.04 | 0.32, 51.06 |
| 3136.47 – 5645.01 | 24/37 | 0.60 | 0.14, 2.56 | 7/16 | 2.69 | 0.23, 32.01 |
| 5645.02 – 21569.07 | 23/35 | 1.39 | 0.32, 5.99 | 13/19 | 5.68 | 0.56, 57.97 |
| PFOS |  |  |  |  |  |  |
| 9.13 – 2058.09 | 27/40 | --- | --- | 3/14 | --- | --- |
| 2058.10 – 5822.69 | 15/41 | 0.33 | 0.07, 1.57 | 7/13 | 10.68 | 0.87, 131.72 |
| 5822.70 – 11481.99 | 21/35 | 0.91 | 0.24, 3.36 | 10/19 | 3.73 | 0.26, 53.38 |
| 11482.0 – 43719.92 | 28/34 | 0.99 | 0.19, 5.25 | 13/20 | 9.80 | 0.87, 110.29 |
| **Detect/Non-detect** |  |  |  |  |  |  |
| PFPeA | 87/139 | 0.75 | 0.11, 5.22 | 32/65 | 6.22 | 0.92, 41.97 |
| PFHxS | 92/152 | 1.86 | 0.53, 6.51 | 33/67 | 3.65 | 0.74, 18.07 |
| 6:2 FTS | 75/133 | 0.87 | 0.23, 3.73 | 33/60 | 0.30 | 0.05, 2.05 |
| PFOSA | 91/151 | 1.37 | 0.42, 4.53 | 33/67 | 3.15 | 0.66, 15.01 |

^1^ Fit using a generalized linear model, with inverse-probability weighting and survey design-based standard errors.

^2^ Quartiles based on the distribution of controls.

| **Supplemental Table S8. Age at Diagnosis-Stratified Odds Ratios (ORs) and 95% Confidence Intervals (CI) for Associations between PFAS Measures in Dried Blood Spots and Childhood Acute Lymphoblastic Leukemia (ALL) in Los Angeles County, California (2000-2015).** | | | | | | |
| --- | --- | --- | --- | --- | --- | --- |
|  | **Cases ≤ 2 years old** | | | **Cases > 2 years old** | | |
| **Chemical (pg/g dried blood)^1^** | **n cases/ controls** | **OR** | **95% CI** | **n cases/ controls** | **OR** | **95% CI** |
| **Continuous** | | | | | | |
| Log2 PFOA | 53/214 | 1.09 | 0.79, 1.52 | 68/214 | 1.22 | 0.74, 2.02 |
| Log2 PFOS | 54/216 | 1.00 | 0.72, 1.38 | 70/216 | 1.25 | 0.85, 1.85 |
| **Categorical ^2^** | | | | | | |
| PFOA |  |  |  |  |  |  |
| 2.14 – 1365.89 | 14/54 | --- | --- | 11/54 | --- | --- |
| 1365.89 – 3136.46 | 13/53 | 1.89 | 0.30, 11.66 | 17/53 | 0.72 | 0.11, 4.63 |
| 3136.47 – 5645.01 | 10/53 | 0.86 | 0.15, 4.80 | 21/53 | 0.80 | 0.17, 3.77 |
| 5645.02 – 21569.07 | 17/54 | 1.65 | 0.29, 9.28 | 19/54 | 1.60 | 0.33, 7.77 |
| PFOS |  |  |  |  |  |  |
| 9.13 – 2058.09 | 15/54 | --- | --- | 15/54 | --- | --- |
| 2058.10 – 5822.69 | 15/54 | 1.30 | 0.29, 5.82 | 7/54 | 0.12 | 0.03, 0.55 |
| 5822.70 – 11481.99 | 6/54 | 0.62 | 0.07, 5.30 | 25/54 | 2.12 | 0.51 8.84 |
| 11482.0 – 43719.92 | 18/54 | 1.81 | 0.33, 10.05 | 23/54 | 1.27 | 0.27, 5.94 |
| **Detect/Non-detect** |  |  |  |  |  |  |
| PFPeA | 51/204 | 3.54 | 0.85, 14.66 | 68/204 | 1.05 | 0.25, 4.51 |
| PFHxS | 55/219 | 2.12 | 0.54, 8.32 | 70/219 | 1.79 | 0.52, 6.18 |
| 6:2 FTS | 49/193 | 1.32 | 0.30, 5.82 | 59/193 | 0.50 | 0.07, 3.53 |
| PFOSA | 55/218 | 0.91 | 0.19, 4.41 | 69/218 | 2.15 | 0.68, 6.83 |

^1^ Fit using a generalized linear model, with inverse-probability weighting and survey design-based standard errors.

^2^ Quartiles based on the distribution of controls.

| **Supplemental Table S9. Sex-Stratified Odds Ratios (ORs) and 95% Confidence Intervals (CI) for Associations between PFAS Measures in Dried Blood Spots and Childhood Acute Lymphoblastic Leukemia (ALL) in Los Angeles County, California (2000-2015).** | | | | | | |
| --- | --- | --- | --- | --- | --- | --- |
|  | **Male** | | | **Female** | | |
| **Chemical (pg/g dried blood)^1^** | **n cases/ controls** | **OR** | **95% CI** | **n cases/ controls** | **OR** | **95% CI** |
| **Continuous** | | | | | | |
| Log2 PFOA | 64/95 | 1.15 | 0.73, 1.79 | 57/119 | 1.23 | 0.90, 1.69 |
| Log2 PFOS | 67/97 | 1.36 | 0.91, 2.03 | 57/119 | 1.10 | 0.80, 1.51 |
| **Categorical ^2^** | | | | | | |
| PFOA |  |  |  |  |  |  |
| 2.14 – 1365.89 | 13/21 | --- | --- | 12/33 | --- | --- |
| 1365.89 – 3136.46 | 13/25 | 1.01 | 0.03, 1.40 | 16/28 | 9.06 | 1.43, 57.43 |
| 3136.47 – 5645.01 | 19/26 | 2.31 | 0.07, 1.64 | 12/27 | 3.83 | 0.56, 26.18 |
| 5645.02 – 21569.07 | 19/23 | 6.87 | 0.32, 8.25 | 17/31 | 3.16 | 0.52, 19.32 |
| PFOS |  |  |  |  |  |  |
| 9.13 – 2058.09 | 14/25 | --- | --- | 16/29 | --- | --- |
| 2058.10 – 5822.69 | 14/24 | 0.33 | 0.18, 5.57 | 8/30 | 0.75 | 0.12, 4.81 |
| 5822.70 – 11481.99 | 16/26 | 0.91 | 0.35, 15.48 | 15/28 | 1.55 | 0.29, 8.20 |
| 11482.0 – 43719.92 | 23/22 | 0.99 | 1.17, 40.37 | 18/32 | 1.01 | 0.19, 5.44 |
| **Detect/Non-detect** |  |  |  |  |  |  |
| PFPeA | 64/89 | 1.17 | 0.25, 5.49 | 55/115 | 4.09 | 0.80, 20.88 |
| PFHxS | 67/99 | 5.79 | 1.67, 20.10 | 58/120 | 1.53 | 0.29, 8.01 |
| 6:2 FTS | 56/88 | 0.69 | 0.12, 4.06 | 52/105 | 0.57 | 0.11, 2.93 |
| PFOSA | 67/98 | 6.04 | 1.71, 21.30 | 57/120 | 0.81 | 0.23, 2.80 |

^1^ Fit using a generalized linear model, with inverse-probability weighting and survey design-based standard errors.

^2^ Quartiles based on the distribution of controls.

| **Supplemental Table S10. Birth Order-Stratified Odds Ratios (ORs) and 95% Confidence Intervals (CI) for Associations between PFAS Measures in Dried Blood Spots and Childhood Acute Lymphoblastic Leukemia (ALL) in Los Angeles County, California (2000-2015).** | | | | | | |
| --- | --- | --- | --- | --- | --- | --- |
|  | **First-born** | | | **Not First-born** | | |
| **Chemical (pg/g dried blood)^1^** | **n cases/ controls** | **OR** | **95% CI** | **n cases/ controls** | **OR** | **95% CI** |
| **Continuous** | | | | | | |
| Log2 PFOA | 52/87 | 1.08 | 0.63, 1.85 | 69/127 | 1.15 | 0.83, 1.59 |
| Log2 PFOS | 53/86 | 1.31 | 0.81, 2.12 | 71/130 | 0.99 | 0.71, 1.38 |
| **Categorical ^2^** | | | | | | |
| PFOA |  |  |  |  |  |  |
| 2.14 – 1365.89 | 9/19 | --- | --- | 16/35 | --- | --- |
| 1365.89 – 3136.46 | 11/25 | 1.39 | 0.12, 6.41 | 18/28 | 1.50 | 0.24, 9.52 |
| 3136.47 – 5645.01 | 12/18 | 2.50 | 0.04, 2.78 | 19/35 | 1.75 | 0.25, 12.21 |
| 5645.02 – 21569.07 | 20/25 | 3.79 | 0.24, 11.41 | 16/29 | 1.45 | 0.27, 7.77 |
| PFOS |  |  |  |  |  |  |
| 9.13 – 2058.09 | 9/22 | --- | --- | 21/32 | --- | --- |
| 2058.10 – 5822.69 | 10/20 | 0.33 | 0.17, 11.23 | 12/34 | 0.28 | 0.05, 1.45 |
| 5822.70 – 11481.99 | 12/23 | 0.91 | 0.30, 20.56 | 19/31 | 1.38 | 0.28, 6.85 |
| 11482.0 – 43719.92 | 22/21 | 0.99 | 0.46, 31.27 | 19/33 | 0.74 | 0.13, 4.31 |
| **Detect/Non-detect** |  |  |  |  |  |  |
| PFPeA | 50/82 | 5.08 | 1.28, 20.20 | 69/122 | 0.14 | 0.02, 0.91 |
| PFHxS | 53/88 | 5.07 | 0.96, 26.67 | 72/131 | 1.01 | 0.21, 4.74 |
| 6:2 FTS | 47/77 | 0.29 | 0.06, 1.27 | 61/116 | 2.00 | 0.46, 8.75 |
| PFOSA | 53/88 | 5.95 | 1.47, 24.08 | 71/130 | 0.30 | 0.06, 1.45 |

^1^ Fit using a generalized linear model, with inverse-probability weighting and survey design-based standard errors.

^2^ Quartiles based on the distribution of controls.

| **Supplemental Table S11. Birthplace-Stratified Odds Ratios (ORs) and 95% Confidence Intervals (CI) for Associations between PFAS Measures in Dried Blood Spots and Childhood Acute Lymphoblastic Leukemia (ALL) in Los Angeles County, California (2000-2015).** | | | | | | |
| --- | --- | --- | --- | --- | --- | --- |
|  | **US-born** | | | **Foreign-born** | | |
| **Chemical (pg/g dried blood)^1^** | **n cases/ controls** | **OR** | **95% CI** | **n cases/ controls** | **OR** | **95% CI** |
| **Continuous** | | | | | | |
| Log2 PFOA | 74/135 | 1.28 | 0.84, 1.96 | 47/79 | 1.05 | 0.67, 1.65 |
| Log2 PFOS | 74/135 | 1.26 | 0.79, 2.02 | 50/81 | 0.98 | 0.63, 1.54 |
| **Categorical ^2^** | | | | | | |
| PFOA |  |  |  |  |  |  |
| 2.14 – 1365.89 | 16/28 | --- | --- | 9/26 | --- | --- |
| 1365.89 – 3136.46 | 16/37 | 1.52 | 0.25, 9.41 | 13/16 | 1.39 | 0.25, 7.83 |
| 3136.47 – 5645.01 | 17/35 | 1.30 | 0.21, 8.02 | 14/18 | 0.94 | 0.17, 5.16 |
| 5645.02 – 21569.07 | 25/35 | 3.09 | 0.61, 15.72 | 11/19 | 1.69 | 0.15, 19.22 |
| PFOS |  |  |  |  |  |  |
| 9.13 – 2058.09 | 18/30 | --- | --- | 12/24 | --- | --- |
| 2058.10 – 5822.69 | 9/34 | 0.21 | 0.04, 1.12 | 13/20 | 1.26 | 0.23, 7.07 |
| 5822.70 – 11481.99 | 20/36 | 2.73 | 0.57, 13.18 | 11/18 | 0.81 | 0.16, 4.24 |
| 11482.0 – 43719.92 | 27/35 | 1.75 | 0.35, 8.75 | 14/19 | 2.07 | 0.16, 27.73 |
| **Detect/Non-detect** |  |  |  |  |  |  |
| PFPeA | 70/128 | 0.69 | 0.10, 4.68 | 49/76 | 2.93 | 0.56, 15.38 |
| PFHxS | 74/138 | 2.39 | 0.66, 8.60 | 51/81 | 2.04 | 0.42, 10.06 |
| 6:2 FTS | 63/121 | 0.81 | 0.17, 3.96 | 45/72 | 1.37 | 0.23, 8.36 |
| PFOSA | 74/137 | 2.86 | 0.80, 10.21 | 50/81 | 2.26 | 0.26, 19.38 |

^1^ Fit using a generalized linear model, with inverse-probability weighting and survey design-based standard errors.

^2^ Quartiles based on the distribution of controls.

| **Supplemental Table S12. Ethnicity-Stratified Odds Ratios (ORs) and 95% Confidence Intervals (CI) for Associations between Non-targeted PFAS Measures in Dried Blood Spots and Childhood Acute Lymphoblastic Leukemia (ALL) in Los Angeles County, California (2000-2015)** | | | | | | |
| --- | --- | --- | --- | --- | --- | --- |
|  | **Hispanic** | | | **Non-Hispanic** | | |
| **Chemical (ng/g dried blood)^1^** | **n cases/ controls** | **OR** | **95% CI** | **n cases/ controls** | **OR** | **95% CI** |
| **Continuous** | | | | | | |
| Log2 C4HF7O3 | 78/132 | 0.94 | 0.62, 1.43 | 28/56 | 2.45 | 1.04, 5.80 |
| Log2 C4HF9O4S | 78/123 | 0.85 | 0.60, 1.19 | 29/59 | 1.42 | 0.82, 2.46 |
| Log2 C5HF9O3 | 69/115 | 0.98 | 0.57, 1.69 | 26/58 | 1.81 | 0.90, 3.64 |
| Log2 C9H2F16O2 | 74/114 | 0.80 | 0.40, 1.59 | 27/50 | 3.37 | 1.30, 8.76 |
| Log2 C14H2F26O2 | 64/80 | 2.06 | 0.85, 5.01 | 18/45 | 1.47 | 0.39, 5.57 |
| Log2 C15H2F28O2 | 65/75 | 1.77 | 0.75, 4.21 | 17/39 | 1.34 | 0.37, 4.83 |
| Log2 C13H2F24O2 | 51/75 | 1.19 | 0.41, 3.49 | 15/34 | 2.65 | 0.47, 15.06 |
| Log2 C10HF19O5 | 45/73 | 0.85 | 0.48, 1.49 | 20/34 | 1.57 | 0.92, 2.67 |
| Log2 C16H8Cl2F6N2O2S | 79/127 | 0.99 | 0.67, 1.46 | 28/58 | 2.09 | 1.02, 4.29 |
| **Categorical ^2^** | | | | | | |
| C4HF7O3 |  |  |  |  |  |  |
| 0.51 - 13.56 | 22/39 | --- | --- | 2/8 | --- | --- |
| 13.57 - 25.82 | 19/32 | 0.72 | 0.17, 3.04 | 4/15 | 5.29 | 0.34, 82.94 |
| 25.83 - 43.83 | 21/32 | 1.08 | 0.19, 6.32 | 12/15 | 9.65 | 1.03, 90.58 |
| 43.84 - 495.28 | 16/29 | 0.62 | 0.08, 4.53 | 10/18 | 16.90 | 1.62, 176.01 |
| C4HF9O4S |  |  |  |  |  |  |
| 2.22 - 60.20 | 26/33 | --- | --- | 5/13 | --- | --- |
| 60.21 - 119.84 | 21/35 | 0.51 | 0.13, 2.08 | 10/10 | 13.59 | 0.72, 255.40 |
| 119.85 - 215.86 | 19/25 | 2.03 | 0.42, 9.90 | 9/20 | 4.06 | 0.28, 57.80 |
| 215.87 - 3530.00 | 12/30 | 0.26 | 0.03, 2.43 | 5/16 | 2.45 | 0.18, 32.85 |
| C5HF9O3 |  |  |  |  |  |  |
| 0.16 - 6.85 | 19/32 | --- | --- | 3/11 | --- | --- |
| 6.86 - 12.06 | 20/26 | 0.38 | 0.08, 1.72 | 5/17 | 3.27 | 0.14, 75.43 |
| 12.07 - 22.28 | 18/30 | 0.53 | 0.08, 3.49 | 12/14 | 14.39 | 1.35, 152.99 |
| 22.29 - 195.08 | 12/27 | 0.73 | 0.13, 4.10 | 6/16 | 2.85 | 0.19, 43.75 |
| C9H2F16O2 |  |  |  |  |  |  |
| 3.39 - 14.74 | 20/31 | --- | --- | 5/10 | --- | --- |
| 14.75 - 23.66 | 24/28 | 0.45 | 0.08, 2.43 | 6/13 | 1.15 | 0.14, 9.72 |
| 23.67 - 37.14 | 18/27 | 0.93 | 0.19, 4.62 | 8/14 | 0.80 | 0.06, 10.13 |
| 37.15 - 426.20 | 12/28 | 0.47 | 0.08, 2.69 | 8/13 | 9.28 | 1.20, 71.63 |
| C14H2F26O2 |  |  |  |  |  |  |
| 2.60 - 23.78 | 7/18 | --- | --- | 3/14 | --- | --- |
| 23.79 - 32.95 | 13/19 | 2.94 | 0.31, 27.62 | 3/11 | 17.82 | 0.45, 700.58 |
| 32.96 - 46.17 | 19/23 | 7.10 | 0.65, 77.38 | 5/9 | 4.89 | 0.29, 83.31 |
| 46.18 - 275.22 | 25/20 | 13.04 | 1.20, 141.2 | 7/11 | 10.05 | 0.91, 110.33 |
| C15H2F28O2 |  |  |  |  |  |  |
| 2.20 - 20.62 | 13/15 | --- | --- | 3/14 | --- | --- |
| 20.63 - 29.37 | 12/18 | 0.66 | 0.12, 3.80 | 5/10 | 22.41 | 1.68, 298.14 |
| 29.38 - 39.47 | 13/21 | 1.23 | 0.19, 7.95 | 5/7 | 119.51 | 8.31, 1718.01 |
| 39.48 - 143.21 | 27/21 | 3.33 | 0.65, 17.05 | 4/8 | 2.77 | 0.19, 40.01 |
| C13H2F24O2 |  |  |  |  |  |  |
| 10.73 - 29.83 | 8/19 | --- | --- | 3/9 | --- | --- |
| 29.84 - 41.80 | 11/14 | 0.50 | 0.04, 6.81 | 4/12 | 10.95 | 0.72, 167.33 |
| 41.81 - 53.56 | 8/21 | 0.87 | 0.10, 7.43 | 0/7 | 0.00 | 0.00, 0.00 |
| 53.57 - 212.81 | 24/21 | 1.70 | 0.18, 16.14 | 8/6 | 6.38 | 0.44, 91.58 |
| C10HF19O5 |  |  |  |  |  |  |
| 0.49 – 3.74 | 17/19 | --- | --- | 1/8 | --- | --- |
| 3.75 – 9.44 | 7/17 | 0.06 | 0.01, 0.57 | 2/10 | 10.99 | 0.60, 202.68 |
| 9.45 – 25.54 | 12/20 | 0.12 | 0.02, 1.00 | 5/6 | 97.92 | 1.26, 7619.50 |
| 25.55 - 516.73 | 9/17 | 1.58 | 0.25, 9.97 | 12/10 | 348.7 | 11.47, 10597.4 |
| C16H8Cl2F6N2O2S |  |  |  |  |  |  |
| 3834 – 60541 | 17/34 | --- | --- | 4/13 | --- | --- |
| 60542 – 119314 | 16/35 | 2.64 | 0.37, 18.99 | 5/11 | 2.50 | 0.22, 28.50 |
| 119315 – 221888 | 29/29 | 2.63 | 0.50, 13.92 | 9/17 | 60.62 | 5.38, 683.63 |
| 221889 – 946875 | 17/29 | 0.99 | 0.18, 5.57 | 10/17 | 19.58 | 1.91, 200.20 |

^1^ Fit using a generalized linear model, with inverse-probability weighting and survey design-based standard errors.

^2^ Quartiles based on the distribution of controls.

| **Supplemental Table S13. Age at Diagnosis-Stratified Odds Ratios (ORs) and 95% Confidence Intervals (CI) for Associations between Non-targeted PFAS Measures in Dried Blood Spots and Childhood Acute Lymphoblastic Leukemia (ALL) in Los Angeles County, California (2000-2015).** | | | | | | |
| --- | --- | --- | --- | --- | --- | --- |
|  | **Cases ≤ 2 years old** | | | **Cases > 2 years old** | | |
| **Chemical (ng/g dried blood)^1^** | **n cases/ controls** | **OR** | **95% CI** | **n cases/ controls** | **OR** | **95% CI** |
| **Continuous** | | | | | | |
| Log2 C4HF7O3 | 45/188 | 1.99 | 0.94, 4.23 | 61/188 | 0.99 | 0.63, 1.56 |
| Log2 C4HF9O4S | 46/182 | 1.11 | 0.70, 1.77 | 61/182 | 1.00 | 0.70, 1.43 |
| Log2 C5HF9O3 | 41/173 | 1.46 | 0.77, 2.79 | 54/173 | 1.17 | 0.69, 1.98 |
| Log2 C9H2F16O2 | 44/164 | 0.96 | 0.39, 2.36 | 57/164 | 1.46 | 0.83, 2.58 |
| Log2 C14H2F26O2 | 37/125 | 1.99 | 0.73, 5.44 | 45/125 | 1.32 | 0.53, 3.25 |
| Log2 C15H2F28O2 | 35/114 | 2.38 | 1.11, 5.10 | 47/114 | 1.12 | 0.46, 2.71 |
| Log2 C13H2F24O2 | 29/109 | 0.99 | 0.27, 3.67 | 37/109 | 0.97 | 0.32, 2.96 |
| Log2 C10HF19O5 | 23/107 | 1.40 | 0.82, 2.40 | 42/107 | 1.18 | 0.81, 1.72 |
| Log2 C16H8Cl2F6N2O2S | 44/185 | 1.45 | 0.88, 2.39 | 63/185 | 1.05 | 0.67, 1.62 |
| **Categorical ^2^** | | | | | | |
| C4HF7O3 |  |  |  |  |  |  |
| 0.51 - 13.56 | 8/47 | --- | --- | 16/47 | --- | --- |
| 13.57 - 25.82 | 11/47 | 2.02 | 0.21, 19.07 | 12/47 | 0.93 | 0.19, 4.58 |
| 25.83 - 43.83 | 16/47 | 2.15 | 0.20, 23.22 | 17/47 | 1.35 | 0.24, 7.55 |
| 43.84 - 495.28 | 10/47 | 6.06 | 0.53, 69.13 | 16/47 | 0.73 | 0.12, 4.42 |
| C4HF9O4S |  |  |  |  |  |  |
| 2.22 - 60.20 | 9/46 | --- | --- | 22/46 | --- | --- |
| 60.21 - 119.84 | 13/45 | 1.81 | 0.17, 19.12 | 18/45 | 1.59 | 0.22, 11.30 |
| 119.85 - 215.86 | 19/45 | 6.01 | 0.78, 46.24 | 9/45 | 1.71 | 0.25, 11.90 |
| 215.87 - 3530.00 | 5/46 | 0.80 | 0.09, 7.30 | 12/46 | 0.95 | 0.15, 6.05 |
| C5HF9O3 |  |  |  |  |  |  |
| 0.16 - 6.85 | 10/43 | --- | --- | 12/43 | --- | --- |
| 6.86 - 12.06 | 8/43 | 0.74 | 0.06, 9.18 | 17/43 | 0.90 | 0.14, 5.94 |
| 12.07 - 22.28 | 16/44 | 3.80 | 0.48, 30.22 | 14/44 | 1.27 | 0.20, 8.07 |
| 22.29 - 195.08 | 7/43 | 1.23 | 0.15, 10.17 | 11/43 | 1.27 | 0.22, 7.30 |
| C9H2F16O2 |  |  |  |  |  |  |
| 3.39 - 14.74 | 14/41 | --- | --- | 11/41 | --- | --- |
| 14.75 - 23.66 | 14/41 | 0.43 | 0.07, 2.57 | 16/41 | 0.66 | 0.10, 4.63 |
| 23.67 - 37.14 | 9/41 | 0.14 | 0.03, 0.78 | 17/41 | 2.00 | 0.34, 11.68 |
| 37.15 - 426.20 | 7/41 | 1.39 | 0.24, 7.90 | 13/41 | 1.27 | 0.22, 7.44 |
| C14H2F26O2 |  |  |  |  |  |  |
| 2.60 - 23.78 | 3/32 | --- | --- | 7/32 | --- | --- |
| 23.79 - 32.95 | 8/30 | 28.58 | 3.59, 227.28 | 8/30 | 1.78 | 0.25, 12.55 |
| 32.96 - 46.17 | 11/32 | 15.41 | 1.76, 135.08 | 13/32 | 4.59 | 0.59, 35.66 |
| 46.18 - 275.22 | 15/31 | 50.95 | 5.81, 446.82 | 17/31 | 1.61 | 0.14, 18.21 |
| C15H2F28O2 |  |  |  |  |  |  |
| 2.20 - 20.62 | 6/29 | --- | --- | 10/29 | --- | --- |
| 20.63 - 29.37 | 6/28 | 1.37 | 0.19, 9.68 | 11/28 | 1.80 | 0.32, 10.05 |
| 29.38 - 39.47 | 11/28 | 8.93 | 1.14, 69.66 | 7/28 | 1.12 | 0.17, 7.32 |
| 39.48 - 143.21 | 12/29 | 3.94 | 0.47, 32.78 | 19/29 | 1.21 | 0.19, 7.61 |
| C13H2F24O2 |  |  |  |  |  |  |
| 10.73 - 29.83 | 3/28 | --- | --- | 8/28 | --- | --- |
| 29.84 - 41.80 | 5/26 | 2.35 | 0.13, 42.30 | 10/26 | 1.66 | 0.20, 14.11 |
| 41.81 - 53.56 | 5/28 | 2.21 | 0.19, 25.25 | 3/28 | 0.83 | 0.08, 8.69 |
| 53.57 - 212.81 | 16/27 | 2.20 | 0.14, 33.82 | 16/27 | 0.93 | 0.14, 6.17 |
| C10HF19O5 |  |  |  |  |  |  |
| 0.49 – 3.74 | 7/27 | --- | --- | 11/27 | --- | --- |
| 3.75 – 9.44 | 3/27 | 0.21 | 0.01, 3.14 | 6/27 | 0.20 | 0.02, 1.75 |
| 9.45 – 25.54 | 5/26 | 0.09 | 0.01, 1.17 | 12/26 | 0.88 | 0.06, 11.96 |
| 25.55 - 516.73 | 8/27 | 7.19 | 1.23, 41.88 | 13/27 | 3.72 | 0.61, 22.83 |
| C16H8Cl2F6N2O2S |  |  |  |  |  |  |
| 3834 - 60541 | 8/47 | --- | --- | 13/47 | --- | --- |
| 60542 - 119314 | 7/46 | 14.61 | 1.17, 182.76 | 14/46 | 0.83 | 0.13, 5.22 |
| 119315 - 221888 | 15/46 | 14.24 | 2.08, 97.70 | 23/46 | 2.07 | 0.44, 9.85 |
| 221889 - 946875 | 14/46 | 6.75 | 0.84, 54.50 | 13/46 | 1.01 | 0.20, 5.07 |

^1^ Fit using a generalized linear model, with inverse-probability weighting and survey design-based standard errors.

^2^ Quartiles based on the distribution of controls.

| **Supplemental Table S14. Sex-Stratified Odds Ratios (ORs) and 95% Confidence Intervals (CI) for Associations between Non-targeted PFAS Measures in Dried Blood Spots and Childhood Acute Lymphoblastic Leukemia (ALL) in Los Angeles County, California (2000-2015).** | | | | | | |
| --- | --- | --- | --- | --- | --- | --- |
|  | **Males** | | | **Females** | | |
| **Chemical (ng/g dried blood)^1^** | **n cases/ controls** | **OR** | **95% CI** | **n cases/ controls** | **OR** | **95% CI** |
| **Continuous** | | | | | | |
| Log2 C4HF7O3 | 54/87 | 1.18 | 0.68, 2.03 | 52/101 | 1.12 | 0.60, 2.09 |
| Log2 C4HF9O4S | 57/85 | 0.83 | 0.57, 1.20 | 50/97 | 1.15 | 0.68, 1.94 |
| Log2 C5HF9O3 | 47/80 | 1.09 | 0.61, 1.93 | 48/93 | 1.12 | 0.56, 2.27 |
| Log2 C9H2F16O2 | 51/79 | 1.18 | 0.60, 2.33 | 50/85 | 0.95 | 0.41, 2.16 |
| Log2 C14H2F26O2 | 46/62 | 1.40 | 0.52, 3.72 | 36/63 | 3.50 | 1.09, 11.22 |
| Log2 C15H2F28O2 | 40/57 | 1.31 | 0.43, 3.98 | 42/57 | 1.87 | 0.56, 6.26 |
| Log2 C13H2F24O2 | 34/55 | 0.81 | 0.24, 2.77 | 32/54 | 2.15 | 0.49, 9.50 |
| Log2 C10HF19O5 | 38/47 | 1.11 | 0.65, 1.89 | 27/60 | 0.93 | 0.49, 1.75 |
| Log2 C16H8Cl2F6N2O2S | 57/84 | 1.27 | 0.79, 2.57 | 50/101 | 1.02 | 0.66, 1.57 |
| **Categorical ^2^** | | | | | | |
| C4HF7O3 |  |  |  |  |  |  |
| 0.51 - 13.56 | 14/18 | --- | --- | 10/29 | --- | --- |
| 13.57 - 25.82 | 11/20 | 0.73 | 0.11, 4.85 | 12/27 | 1.29 | 0.17, 9.65 |
| 25.83 - 43.83 | 13/22 | 1.86 | 0.18, 18.80 | 20/25 | 1.23 | 0.23, 6.45 |
| 43.84 - 495.28 | 16/27 | 1.18 | 0.12, 11.80 | 10/20 | 1.59 | 0.18, 14.24 |
| C4HF9O4S |  |  |  |  |  |  |
| 2.22 - 60.20 | 20/19 | --- | --- | 11/27 | --- | --- |
| 60.21 - 119.84 | 15/19 | 0.41 | 0.09, 1.96 | 16/26 | 3.32 | 0.37, 29.59 |
| 119.85 - 215.86 | 13/22 | 1.43 | 0.24, 8.36 | 15/23 | 3.35 | 0.25, 44.24 |
| 215.87 - 3530.00 | 9/25 | 0.15 | 0.02, 1.37 | 8/21 | 1.74 | 0.14, 20.93 |
| C5HF9O3 |  |  |  |  |  |  |
| 0.16 - 6.85 | 11/16 | --- | --- | 11/27 | --- | --- |
| 6.86 - 12.06 | 12/18 | 1.15 | 0.12, 10.99 | 13/25 | 0.39 | 0.05, 3.32 |
| 12.07 - 22.28 | 13/22 | 1.10 | 0.11, 11.22 | 17/22 | 2.88 | 0.37, 22.53 |
| 22.29 - 195.08 | 11/24 | 0.47 | 0.04, 5.15 | 7/19 | 0.92 | 0.11, 7.82 |
| C9H2F16O2 |  |  |  |  |  |  |
| 3.39 - 14.74 | 14/12 | --- | --- | 11/29 | --- | --- |
| 14.75 - 23.66 | 10/28 | 0.05 | 0.01, 0.31 | 20/13 | 5.06 | 1.02, 25.23 |
| 23.67 - 37.14 | 15/18 | 0.41 | 0.06, 2.92 | 11/23 | 0.55 | 0.05, 6.38 |
| 37.15 - 426.20 | 12/21 | 0.54 | 0.10, 2.92 | 8/20 | 1.26 | 0.15, 10.48 |
| C14H2F26O2 |  |  |  |  |  |  |
| 2.60 - 23.78 | 3/12 | --- | --- | 7/20 | --- | --- |
| 23.79 - 32.95 | 11/22 | 1.91 | 0.07, 53.93 | 5/8 | 3.03 | 0.33, 27.63 |
| 32.96 - 46.17 | 12/13 | 5.46 | 0.21, 141.04 | 12/19 | 7.56 | 0.83, 69.02 |
| 46.18 - 275.22 | 20/15 | 4.65 | 0.15, 149.04 | 12/16 | 20.52 | 1.67, 252.34 |
| C15H2F28O2 |  |  |  |  |  |  |
| 2.20 - 20.62 | 9/10 | --- | --- | 7/19 | --- | --- |
| 20.63 - 29.37 | 6/18 | 0.08 | 0.01, 0.89 | 11/10 | 16.16 | 2.33, 112.03 |
| 29.38 - 39.47 | 7/15 | 0.34 | 0.03, 3.48 | 11/13 | 20.24 | 2.59, 158.28 |
| 39.48 - 143.21 | 18/14 | 0.80 | 0.10, 6.27 | 13/15 | 4.79 | 0.42, 54.86 |
| C13H2F24O2 |  |  |  |  |  |  |
| 10.73 - 29.83 | 5/14 | --- | --- | 6/14 | --- | --- |
| 29.84 - 41.80 | 9/15 | 1.29 | 0.09, 18.22 | 6/11 | 4.19 | 0.27, 64.13 |
| 41.81 - 53.56 | 5/16 | 1.25 | 0.14, 11.17 | 3/12 | 0.51 | 0.04, 6.79 |
| 53.57 - 212.81 | 15/10 | 0.99 | 0.08, 12.06 | 17/17 | 3.72 | 0.37, 36.93 |
| C10HF19O5 |  |  |  |  |  |  |
| 0.49 – 3.74 | 12/6 | --- | --- | 6/21 | --- | --- |
| 3.75 – 9.44 | 4/14 | 0.17 | 0.01, 4.07 | 5/13 | 0.20 | 0.01, 2.75 |
| 9.45 – 25.54 | 10/14 | 0.22 | 0.01, 5.31 | 7/12 | 0.12 | 0.01, 1.23 |
| 25.55 - 516.73 | 12/13 | 1.60 | 0.08, 31.71 | 9/14 | 6.06 | 0.53, 69.21 |
| C16H8Cl2F6N2O2S |  |  |  |  |  |  |
| 3834 – 60541 | 11/16 | --- | --- | 10/31 | --- | --- |
| 60542 – 119314 | 11/23 | 2.60 | 0.20, 34.52 | 10/23 | 3.95 | 0.45, 34.37 |
| 119315 – 221888 | 24/22 | 5.59 | 0.69, 45.46 | 14/24 | 2.55 | 0.42, 15.53 |
| 221889 – 946875 | 11/23 | 5.37 | 0.58, 49.49 | 16/23 | 0.89 | 0.18, 4.44 |

^1^ Fit using a generalized linear model, with inverse-probability weighting and survey design-based standard errors.

^2^ Quartiles based on the distribution of controls.

| **Supplemental Table S15. Birth Order-Stratified Odds Ratios (ORs) and 95% Confidence Intervals (CI) for Associations between Non-targeted PFAS Measures in Dried Blood Spots and Childhood Acute Lymphoblastic Leukemia (ALL) in Los Angeles County, California (2000-2015).** | | | | | | |
| --- | --- | --- | --- | --- | --- | --- |
|  | **First-born** | | | **Not First-born** | | |
| **Chemical (ng/g dried blood)^1^** | **n cases/ controls** | **OR** | **95% CI** | **n cases/ controls** | **OR** | **95% CI** |
| **Continuous** | | | | | | |
| Log2 C4HF7O3 | 46/76 | 1.41 | 0.77, 2.56 | 60/112 | 0.91 | 0.51, 1.63 |
| Log2 C4HF9O4S | 46/71 | 1.17 | 0.77, 1.76 | 61/111 | 1.03 | 0.63, 1.68 |
| Log2 C5HF9O3 | 42/65 | 1.41 | 0.74, 2.70 | 53/108 | 0.99 | 0.54, 1.82 |
| Log2 C9H2F16O2 | 43/64 | 1.16 | 0.57, 2.39 | 58/100 | 1.12 | 0.48, 2.61 |
| Log2 C14H2F26O2 | 37/48 | 0.73 | 0.16, 3.41 | 45/77 | 3.74 | 1.76, 7.95 |
| Log2 C15H2F28O2 | 35/43 | 1.09 | 0.25, 4.67 | 47/71 | 2.12 | 1.07, 4.21 |
| Log2 C13H2F24O2 | 32/39 | 0.69 | 0.14, 3.33 | 34/70 | 1.38 | 0.44, 4.30 |
| Log2 C10HF19O5 | 30/45 | 2.06 | 0.96, 4.43 | 35/62 | 0.68 | 0.37, 1.23 |
| Log2 C16H8Cl2F6N2O2S | 44/74 | 1.45 | 0.84, 2.52 | 63/111 | 0.94 | 0.58, 1.52 |
| **Categorical ^2^** | | | | | | |
| C4HF7O3 |  |  |  |  |  |  |
| 0.51 - 13.56 | 12/21 | --- | --- | 12/26 | --- | --- |
| 13.57 - 25.82 | 7/20 | 0.43 | 0.04, 4.58 | 16/27 | 2.19 | 0.26, 18.21 |
| 25.83 - 43.83 | 11/15 | 1.16 | 0.13, 10.56 | 22/32 | 1.52 | 0.19, 11.94 |
| 43.84 - 495.28 | 16/20 | 1.69 | 0.18, 15.83 | 10/27 | 1.00 | 0.12, 8.76 |
| C4HF9O4S |  |  |  |  |  |  |
| 2.22 - 60.20 | 13/19 | --- | --- | 18/27 | --- | --- |
| 60.21 - 119.84 | 10/19 | 1.20 | 0.15, 9.62 | 21/26 | 2.61 | 0.23, 29.59 |
| 119.85 - 215.86 | 14/15 | 7.35 | 1.11, 48.81 | 14/30 | 1.83 | 0.17, 20.22 |
| 215.87 - 3530.00 | 9/18 | 0.77 | 0.07, 8.11 | 8/28 | 2.64 | 0.35, 19.80 |
| C5HF9O3 |  |  |  |  |  |  |
| 0.16 - 6.85 | 12/16 | --- | --- | 10/27 | --- | --- |
| 6.86 - 12.06 | 6/12 | 0.72 | 0.05, 10.86 | 19/31 | 1.03 | 0.11, 9.27 |
| 12.07 - 22.28 | 13/20 | 0.90 | 0.09, 8.64 | 17/24 | 1.38 | 0.15, 12.85 |
| 22.29 - 195.08 | 11/17 | 0.99 | 0.08, 11.98 | 7/26 | 1.39 | 0.21, 9.25 |
| C9H2F16O2 |  |  |  |  |  |  |
| 3.39 - 14.74 | 12/17 | --- | --- | 13/24 | --- | --- |
| 14.75 - 23.66 | 8/17 | 0.35 | 0.03, 4.19 | 22/24 | 0.47 | 0.06, 3.48 |
| 23.67 - 37.14 | 11/9 | 0.35 | 0.02, 5.03 | 15/32 | 1.13 | 0.18, 7.08 |
| 37.15 - 426.20 | 12/21 | 0.64 | 0.07, 5.71 | 8/20 | 0.95 | 0.14, 6.41 |
| C14H2F26O2 |  |  |  |  |  |  |
| 2.60 - 23.78 | 5/13 | --- | --- | 5/19 | --- | --- |
| 23.79 - 32.95 | 4/11 | 4.46 | 0.22, 89.71 | 12/19 | 5.35 | 0.70, 41.12 |
| 32.96 - 46.17 | 13/10 | 3.19 | 0.18, 56.10 | 11/22 | 8.12 | 1.09, 60.48 |
| 46.18 - 275.22 | 15/14 | 1.29 | 0.05, 36.83 | 17/17 | 60.25 | 10.35, 350.64 |
| C15H2F28O2 |  |  |  |  |  |  |
| 2.20 - 20.62 | 5/14 | --- | --- | 11/15 | --- | --- |
| 20.63 - 29.37 | 7/7 | 4.23 | 0.18, 99.31 | 10/21 | 0.65 | 0.09, 4.88 |
| 29.38 - 39.47 | 9/11 | 4.62 | 0.27, 79.01 | 9/17 | 2.16 | 0.26, 17.70 |
| 39.48 - 143.21 | 14/11 | 1.89 | 0.11, 33.87 | 17/18 | 2.93 | 0.50, 17.33 |
| C13H2F24O2 |  |  |  |  |  |  |
| 10.73 - 29.83 | 4/10 | --- | --- | 7/18 | --- | --- |
| 29.84 - 41.80 | 7/9 | 1.45 | 0.05, 38.66 | 8/17 | 0.82 | 0.05, 12.62 |
| 41.81 - 53.56 | 4/7 | 1.09 | 0.06, 19.70 | 4/21 | 0.12 | 0.01, 1.54 |
| 53.57 - 212.81 | 17/13 | 1.47 | 0.07, 31.50 | 15/14 | 1.61 | 0.10, 24.91 |
| C10HF19O5 |  |  |  |  |  |  |
| 0.49 – 3.74 | 7/14 | --- | --- | 11/13 | --- | --- |
| 3.75 – 9.44 | 2/10 | 4.03 | 0.05, 330.86 | 7/17 | 0.05 | 0.01, 0.39 |
| 9.45 – 25.54 | 8/13 | 0.88 | 0.01, 111.10 | 9/13 | 0.36 | 0.05, 2.60 |
| 25.55 - 516.73 | 13/8 | 52.28 | 2.45, 1116.08 | 8/19 | 0.35 | 0.03, 3.75 |
| C16H8Cl2F6N2O2S |  |  |  |  |  |  |
| 3834 – 60541 | 9/17 | --- | --- | 12/30 | --- | --- |
| 60542 – 119314 | 8/18 | 0.51 | 0.07, 3.82 | 13/28 | 13.75 | 0.98, 192.84 |
| 119315 – 221888 | 14/22 | 5.38 | 1.08, 26.79 | 24/24 | 4.06 | 0.46, 35.77 |
| 221889 – 946875 | 13/17 | 0.72 | 0.13, 3.95 | 14/29 | 4.84 | 0.74, 31.43 |

^1^ Fit using a generalized linear model, with inverse-probability weighting and survey design-based standard errors.

^2^ Quartiles based on the distribution of controls.

| **Supplemental Table S16. Birthplace-Stratified Odds Ratios (ORs) and 95% Confidence Intervals (CI) for Associations between Non-targeted PFAS Measures in Dried Blood Spots and Childhood Acute Lymphoblastic Leukemia (ALL) in Los Angeles County, California (2000-2015).** | | | | | | |
| --- | --- | --- | --- | --- | --- | --- |
|  | **US-born** | | | **Foreign-born** | | |
| **Chemical (ng/g dried blood)^1^** | **n cases/ controls** | **OR** | **95% CI** | **n cases/ controls** | **OR** | **95% CI** |
| **Continuous** | | | | | | |
| Log2 C4HF7O3 | 65/118 | 1.11 | 0.70, 1.76 | 41/70 | 1.34 | 0.72, 2.51 |
| Log2 C4HF9O4S | 61/117 | 1.20 | 0.77, 1.87 | 46/65 | 0.90 | 0.62, 1.31 |
| Log2 C5HF9O3 | 59/111 | 1.20 | 0.59, 2.44 | 36/62 | 1.05 | 0.57, 1.93 |
| Log2 C9H2F16O2 | 63/103 | 1.15 | 0.50, 2.68 | 38/61 | 1.14 | 0.51, 2.50 |
| Log2 C14H2F26O2 | 47/80 | 1.03 | 0.33, 3.20 | 35/45 | 1.66 | 0.57, 4.86 |
| Log2 C15H2F28O2 | 49/69 | 1.02 | 0.37, 2.81 | 33/45 | 3.21 | 1.14, 9.03 |
| Log2 C13H2F24O2 | 39/68 | 0.84 | 0.21, 3.31 | 27/41 | 0.73 | 0.15, 3.57 |
| Log2 C10HF19O5 | 39/68 | 1.21 | 0.65, 2.25 | 26/39 | 1.24 | 0.81, 1.89 |
| Log2 C16H8Cl2F6N2O2S | 64/120 | 1.06 | 0.66, 1.71 | 43/65 | 1.43 | 0.84, 2.42 |
| **Categorical ^2^** | | | | | | |
| C4HF7O3 |  |  |  |  |  |  |
| 0.51 - 13.56 | 14/24 | --- | --- | 10/23 | --- | --- |
| 13.57 - 25.82 | 16/31 | 1.12 | 0.19, 6.48 | 7/16 | 1.97 | 0.30, 12.69 |
| 25.83 - 43.83 | 22/31 | 1.43 | 0.25, 8.36 | 11/16 | 2.51 | 0.35, 17.91 |
| 43.84 - 495.28 | 13/32 | 0.63 | 0.07, 5.41 | 13/15 | 7.04 | 0.66, 75.38 |
| C4HF9O4S |  |  |  |  |  |  |
| 2.22 - 60.20 | 16/29 | --- | --- | 15/17 | --- | --- |
| 60.21 - 119.84 | 13/30 | 0.62 | 0.08, 4.72 | 18/15 | 3.61 | 0.68, 19.00 |
| 119.85 - 215.86 | 20/26 | 11.47 | 1.93, 68.21 | 8/19 | 1.88 | 0.26, 13.50 |
| 215.87 - 3530.00 | 12/32 | 1.24 | 0.11, 13.69 | 5/14 | 0.54 | 0.07, 3.92 |
| C5HF9O3 |  |  |  |  |  |  |
| 0.16 - 6.85 | 14/24 | --- | --- | 8/19 | --- | --- |
| 6.86 - 12.06 | 17/30 | 0.26 | 0.04, 1.88 | 8/13 | 1.35 | 0.15, 11.92 |
| 12.07 - 22.28 | 15/28 | 1.18 | 0.12, 11.41 | 15/16 | 3.61 | 0.49, 26.63 |
| 22.29 - 195.08 | 13/29 | 0.76 | 0.07, 8.78 | 5/14 | 0.30 | 0.03, 3.06 |
| C9H2F16O2 |  |  |  |  |  |  |
| 3.39 - 14.74 | 18/24 | --- | --- | 7/17 | --- | --- |
| 14.75 - 23.66 | 16/27 | 0.19 | 0.04, 1.01 | 14/14 | 1.09 | 0.18, 6.61 |
| 23.67 - 37.14 | 18/25 | 0.77 | 0.13, 4.75 | 8/16 | 0.54 | 0.07, 4.00 |
| 37.15 - 426.20 | 11/27 | 0.80 | 0.11, 5.89 | 9/14 | 1.49 | 0.18, 12.33 |
| C14H2F26O2 |  |  |  |  |  |  |
| 2.60 - 23.78 | 7/18 | --- | --- | 3/14 | --- | --- |
| 23.79 - 32.95 | 7/16 | 3.07 | 0.19, 49.86 | 9/14 | 22.00 | 1.96, 247.28 |
| 32.96 - 46.17 | 16/23 | 1.79 | 0.11, 30.55 | 8/9 | 91.08 | 4.45, 1865.53 |
| 46.18 - 275.22 | 17/23 | 2.54 | 0.13, 49.63 | 15/8 | 36.16 | 3.20, 408.79 |
| C15H2F28O2 |  |  |  |  |  |  |
| 2.20 - 20.62 | 10/13 | --- | --- | 6/16 | --- | --- |
| 20.63 - 29.37 | 10/16 | 2.54 | 0.26, 24.68 | 7/12 | 4.36 | 0.37, 51.30 |
| 29.38 - 39.47 | 8/22 | 0.17 | 0.02, 1.73 | 10/6 | 140.84 | 7.51, 2640.85 |
| 39.48 - 143.21 | 21/18 | 1.65 | 0.22, 12.68 | 10/11 | 19.87 | 2.12, 186.17 |
| C13H2F24O2 |  |  |  |  |  |  |
| 10.73 - 29.83 | 8/13 | --- | --- | 3/15 | --- | --- |
| 29.84 - 41.80 | 6/17 | 0.07 | 0.01, 0.67 | 9/9 | 18.15 | 1.13, 292.06 |
| 41.81 - 53.56 | 4/19 | 0.11 | 0.01, 2.08 | 4/9 | 16.76 | 1.54, 182.01 |
| 53.57 - 212.81 | 21/19 | 0.41 | 0.05, 3.61 | 11/8 | 4.95 | 0.42, 58.40 |
| C10HF19O5 |  |  |  |  |  |  |
| 0.49 – 3.74 | 13/15 | --- | --- | 5/12 | --- | --- |
| 3.75 – 9.44 | 5/18 | 0.04 | 0.00, 0.84 | 4/9 | 0.70 | 0.05, 10.56 |
| 9.45 – 25.54 | 13/17 | 0.49 | 0.02, 13.90 | 4/9 | 0.08 | 0.01, 0.80 |
| 25.55 - 516.73 | 8/18 | 1.27 | 0.14, 11.88 | 13/9 | 9.04 | 0.86, 95.03 |
| C16H8Cl2F6N2O2S |  |  |  |  |  |  |
| 3834 – 60541 | 13/26 | --- | --- | 8/21 | --- | --- |
| 60542 – 119314 | 10/35 | 1.95 | 0.09, 40.61 | 11/11 | 1.54 | 0.19, 12.24 |
| 119315 – 221888 | 24/31 | 5.07 | 0.67, 38.09 | 14/15 | 2.38 | 0.26, 21.70 |
| 221889 – 946875 | 17/28 | 5.15 | 0.62, 42.62 | 10/18 | 0.59 | 0.09, 4.03 |

^1^ Fit using a generalized linear model, with inverse-probability weighting and survey design-based standard errors.

^2^ Quartiles based on the distribution of controls.
